# LncRNA *HOTAIRM1* functions in DNA double-strand break repair via its association with DNA repair and mRNA surveillance factors

**DOI:** 10.1093/nar/gkad143

**Published:** 2023-03-07

**Authors:** Tzu-Wei Chuang, Chun-Hao Su, Pei-Yu Wu, Yao-Ming Chang, Woan-Yuh Tarn

**Affiliations:** Institute of Biomedical Sciences, Academia Sinica, 128 Academy Road Section 2, Nankang, Taipei 11529, Taiwan; Institute of Biomedical Sciences, Academia Sinica, 128 Academy Road Section 2, Nankang, Taipei 11529, Taiwan; Institute of Biological Chemistry, Academia Sinica, Taipei, Taiwan; Institute of Biomedical Sciences, Academia Sinica, 128 Academy Road Section 2, Nankang, Taipei 11529, Taiwan; Institute of Biomedical Sciences, Academia Sinica, 128 Academy Road Section 2, Nankang, Taipei 11529, Taiwan

## Abstract

The eukaryotic exon junction complex component Y14 participates in double-strand break (DSB) repair via its RNA-dependent interaction with the non-homologous end-joining (NHEJ) complex. Using immunoprecipitation-RNA-seq, we identified a set of Y14-associated long non-coding RNAs (lncRNAs). The lncRNA *HOTAIRM1* serves as a strong candidate that mediates the interaction between Y14 and the NHEJ complex. *HOTAIRM1* localized to near ultraviolet laser-induced DNA damage sites. Depletion of *HOTAIRM1* delayed the recruitment of DNA damage response and repair factors to DNA lesions and compromised the efficiency of NHEJ-mediated DSB repair. Identification of the *HOTAIRM1* interactome revealed a large set of RNA processing factors including mRNA surveillance factors. The surveillance factors Upf1 and SMG6 localized to DNA damage sites in a *HOTAIRM1*-dependent manner. Depletion of Upf1 or SMG6 increased the level of DSB-induced non-coding transcripts at damaged sites, indicating a pivotal role for Upf1/SMG6-mediated RNA degradation in DNA repair. We conclude that *HOTAIRM1* serves as an assembly scaffold for both DNA repair and mRNA surveillance factors that act in concert to repair DSBs.

## INTRODUCTION

DNA damage may arise from physiological processes or result from exposure to genotoxic agents (1). Inefficient repair of DNA lesions threatens genome stability and underlies a number of human diseases, particularly cancer. DNA double-strand break (DSB) is one of the most hazardous DNA lesions. DNA damage triggers the DNA damage response (DDR), which comprises a network of cellular pathways that sense and repair DNA lesions, and activates cell cycle checkpoints to safeguard genome integrity or cause apoptosis (2). The Mre11–Rad50–Nbs1 (MRN) complex acts as a DSB sensor and functions in initial DSB processing and DDR activation. Two principal mechanisms are used for DSB repair, namely non-homologous end-joining (NHEJ) and homologous recombination (HR). NHEJ is considered to be the dominant DSB repair pathway, albeit with a higher error rate. The core repair machinery of NHEJ comprises a set of proteins including the Ku70/80 heterodimer, DNA-dependent protein kinase (DNA-PK) catalytic subunit, the DNA endonuclease Artemis, DNA ligase IV, XRCC4-like factor (XLF) and DNA polymerases λ/μ. The binding of Ku70/80 to DSB ends activates DNA-PK and NHEJ (3).

In addition to canonical DNA repair factors, RNA processing factors are also critical for maintenance of genome stability. Some of these factors control the expression of DNA repair proteins or directly participate in DNA damage responses via interaction with DNA repair factors. For example, BCLAF1 cooperates with phosphorylated BRCA1 to regulate the splicing of the transcripts encoding DNA repair factors after DNA damage (4). SFPQ/PSF promotes HR via its interaction with the Rad51 recombinase and NHEJ by substituting XLF (5,6). Poly(ADP-ribose) polymerase 1 (PARP1) catalyzes the formation of poly(ADP-ribose) onto itself or target proteins immediately after DNA damage (7). Some of the RNA-binding proteins (RBPs) are recruited to DNA damage sites in a poly(ADP-ribose)-dependent manner (4,8,9). Localized RBPs can form liquid phase-separated compartments that facilitate the recruitment of DSB repair factors (10,11). However, exactly how the various RBPs function in DSB repair awaits investigation.

Besides RBPs, RNAs also play a role in DDR or DNA repair (12,13). Indeed, DNA damage-induced long non-coding RNAs (lncRNAs) can regulate gene expression via interactions with DNA/RNA-binding proteins such as p53, TLS and YBX1 (14–16). For example, *Norad* sequesters Pumilio proteins to suppress the expression of mitotic and DNA repair factors, and may form a complex with topoisomerase I to ensure proper cell cycle progression and chromosome segregation (17,18). Furthermore, several different types of ncRNAs directly participate in DSB repair. DSB sites can bidirectionally generate damage-induced lncRNAs (dilncRNAs) through transcription by RNA polymerase II (19,20). The MRN complex facilitates such DSB-induced transcription by melting DNA ends (21). These dilncRNAs are subsequently processed into small RNAs in a Dicer/Drosha-dependent or -independent manner at repetitive regions or ribosomal DNA loci (19,22,23). RNA hybrids formed by these dilncRNAs and small RNAs serve as a signal for recruiting the DDR factors MDC1 and 53BP1 to DNA damage sites (19). The dilncRNAs can also form hybrids with resected single-stranded DNA ends in cell cycle phases S and G_2_ to recruit HR factors (24). Besides the transcripts generated from DSB sites, several lncRNAs have direct roles in DSB repair. *BGL3* and *DDSR1* regulate BRCA1 accumulation at DSBs (21,25). Several other lncRNAs, such as *LINP1* and *SNHG12*, mediate the interaction between DNA-PK and Ku70/80, and hence participate in NHEJ (26,27). The mechanisms underlying the function of individual RNAs in DNA damage repair require further investigation.

Y14/RBM8A functions in mRNA localization in the *Drosophila* germline and acts as a core factor of the exon junction complex (EJC) in higher eukaryotes, which provides a link between splicing and nonsense-mediated decay (NMD) of mRNAs (28). Y14 also modulates alternative splicing of precursor mRNAs, particularly those involved in apoptosis and cell cycle progression. Accordingly, depletion of Y14 causes cell cycle arrest and apoptosis (29,30). Several lines of evidence indicate that Y14 is important for maintenance of genome or chromosome integrity (30–32). We reported that Y14, but not other EJC factors, specifically interacts with the NHEJ and DDR factors (31). Depletion of Y14 causes delayed recruitment of these factors to DSB sites and thus impairs DNA repair. The notion that Y14 interacts with NHEJ factors in an RNA-dependent manner suggests the involvement of RNA in Y14-mediated DNA repair. To test this hypothesis, we identified Y14-associated lncRNAs that participate in the NHEJ pathway and explored the mechanism underlying RNA-mediated DSB repair.

## MATERIALS AND METHODS

### Cell culture and transfection

Cell culture and transient transfection of HeLa, HEK293 and U2OS cells were performed as previously described (31). All small interfering RNAs (siRNAs), antisense oligonucleotides, biotinylated antisense oligonucleotides, antisense GapmerRs and primers are listed in Supplementary Table S8. U2OS cells that stably express green fluorescent protein (GFP)-fused DDR factors (Ku80, Ku70 or MDC1) were used previously (31,33). For DNA damage induction, HeLa cells were irradiated with X-rays (10 Gy).

### Plasmids

The expression vectors encoding FLAG-tagged Y14 (wild type, SA or WV mutant), eIF4A3 and Ku70 were described previously (31). The pLKO.1-sh*HOTAIRM1*-mCherry vector was constructed as previously described for pLKO.1-shY14-mCherry (31); the sh*HOTAIRM1* sequence was: 5′-AAATGTGGGTGTTTGAAACAACTCGAGTTGTTTCAAACACCCACATTT. The pSCE expression vector was provided by the HeLa cell-based NHEJ screening kit (TopoGEN). For CRISPR/Cas9 [clustered regularly interspaced short palindromic repeats (CRISPR)/CRISPR-associated 9 (Cas9)]-mediated DNA cleavage, the pAll-Cas9.Ppuro vector expressing a hypoxanthine guanine phosphoribosyltransferase (HPRT) gene-targeting single guide RNA (sgRNA; 5′-GCAAAGGGTGTTTATTCCTCA-3′) was constructed according to Du *et al.* (34) by the National RNAi Core Facility, Academia Sinica, Taiwan. The *HOTAIRM1* cDNA was chemically synthesized (TOOLS, Taiwan), and inserted into the pcDNA vector. Three stop codons and six MS2-binding sites that were amplified by polymerase chain reaction (PCR) from the β6MS2 reporter (35) were inserted into the 5′ and 3′ ends, respectively, resulting in pcDNA-*HOTAIRM1*-6× MS2. The truncated versions of *HOTAIRM1* (Δ5′, ΔE2 or Δ3′) were generated by using a PCR-based strategy. The pcDNA-MCP-GFP expression vector was described previously (35). The SMG6-expressing vector was a kind gift of Shigeo Ohno and Niels Gehring (36,37). The SMG6-PIN mutant was generated by PCR-based mutagenesis. The sequence of all the resulting constructs was confirmed. The pEGFP-C1-FLAG-Ku70 was purchased from Addgene.

### Chromatin fractionation and immunoprecipitation

HEK293 cells were transiently transfected with an empty vector (as control) or FLAG-tagged Y14. Forty-eight hours post-transfection, cells were washed with phosphate-buffered saline (PBS) and then incubated at 4°C for 3 min in the cytoskeleton (CSK) buffer containing 10 mM PIPES (pH 7.0), 100 mM NaCl, 300 mM sucrose, 3 mM MgCl_2_ and 0.7% Triton X-100. The supernatants were saved as the soluble fraction, including cytosolic and nucleoplasmic fractions. The chromatin-enriched pellet was removed by centrifugation, washed with CSK buffer twice, resuspended in CSK buffer containing 500 mM NaCl and finally incubated at 4°C for 15 min. After centrifugation at 12 000 *g* for 15 min, the supernatant was collected as the chromatin-associated fraction and used for immunoprecipitation. For immunoprecipitation, the lysates were incubated with anti-FLAG M2 affinity gel (Sigma) at 4°C for 2 h. Beads were then washed with NET-2 buffer (150 mM NaCl, 50 mM Tris–HCl and 0.05% NP-40), followed by RNA extraction using TRIzol reagent (Thermo Fisher Scientific) or protein extraction using sodium dodecylsulfate–polyacrylamide gel electrophoresis (SDS–PAGE) sample buffer (31).

### RNA sequencing and analysis

For RNA sequencing, both input and immunoprecipitated RNAs (one set of vec-IP and two sets of Y14-IP) were subjected to quality check and quantification by using the Qubit RNA HS Assay (Thermo Fisher Scientific), and size profiling by using the BioAnalyzer RNA Nano Assay (Agilent). The RNA-seq library was constructed by using SMARTer Stranded RNA Kit-Pico Input Mammalian (Takara Bio USA) following the manufacturer's protocol. In brief, 0.4–10 ng of RNA was subjected to heat fragmentation and first-strand cDNA synthesis with SMARTScribe reverse transcripion enzyme and template-switching oligo (TSO). The cDNA products were then PCR-amplified with simultaneous barcode engineering, and purified by AMPure XP beads (Beckman Coulter). A reaction of the kit's Control RNA was carried out in parallel. The ribosomal cDNA fragments were depleted using the denatured R-probes of the mammalian kit, and the recovery was determined. After AMPure bead purification, the final cDNA libraries were checked by Qubit HS DNA (Thermo Fisher Scientific) and BioA HS DNA Assay (Agilent). The libraries were normalized for effective molar concentrations by quantitative PCR (qPCR) using KAPA Library Quantification Kit Illumina® Platforms (Roche) against the concentration standards. Next-generation sequencing was conducted with SR101nt format (single-end reads, length 101 nt) on a HiSeq 2500 sequencer (Illumina), and 35.4–41.5 million reads per sample were obtained. The data are strand specific due to the cDNA orientation anchored at the TSO step. Sample preparation and sequencing work were conducted at the High Throughput Genomics Core of Academia Sinica, Taiwan.

The processed reads were mapped to the human genome (GRCh38) to estimate the gene expression level using the Tuxedo protocol (38). The NOISeq R package (39) was used to identify Y14-associated transcripts. The correlation coefficient of two duplicate Y14-IP samples was 0.75, indicating substantial reproducibility of the ribonucleoprotein immunoprecipitation (RIP)-seq. The pathway enrichment test for identified transcripts was conducted by WebGestalt (40) with a false discovery rate (FDR) <0.05. From the set of differentially expressed genes, 349 lncRNAs were identified according to the LNCipedia database (41). The RNA-seq data have been deposited in the NCBI SRA database with the BioProject accession ID PRJNA827119, https://www.ncbi.nlm.nih.gov/bioproject/827119.

### UV-cross-linking and immunoprecipitation

HEK293 cells were transiently transfected with an empty vector or FLAG-Y14 (wild type, SA or WV mutant), FLAG-eIF4A3 and GFP–FLAG-Ku70. GapmeRs together with the expression vector of FLAG-Y14 or GFP–FLAG-Ku70 were transfected into HeLa cells. At 48 h post-transfection, cells were UV-cross-linked with 100 mJ/cm^2^ (Stratagene) and lysed in hypotonic buffer containing 10 mM Tris–HCl (pH 7.5), 10 mM NaCl, 10 mM EDTA, 0.5% Triton X-100 and protease inhibitor cocktail (Roche Applied Science) on ice for 10 min. Subsequently, additional NaCl was added to the lysate to a final concentration of 150 mM. After centrifugation at 13 400 *g* at 4°C for 15 min, cell debris was removed, followed by immunoprecipitation using anti-FLAG M2 affinity gel (Sigma). After incubation at 4°C for 2 h, beads were washed with NET-2 buffer. For RNase treatment, washed beads were treated with RNase A (0.2 mg/ml) and incubated at 37°C for 10 min followed by a wash with NET-2 buffer. Immunoprecipitates were subjected to immunoblotting and reverse transcription–PCR (RT–PCR), respectively, for protein and RNA analysis.

### RNase H cleavage

HEK293 cells were UV-cross-linked and lysed in hypotonic buffer as described above. After removal of cell debris, anti-FLAG M2 affinity gel and antisense DNA oligonucleotides (5 μM) were added to the lysates and incubated at 4°C for 2 h. RNase H (50 U/ml, New England BioLabs) digestion was carried out at 37°C for 1 h. The beads were subsequently washed with NET-2 buffer and bound proteins were subjected to immunoblotting.

### RNA pull-down and mass spectrometric analysis

UV-cross-linking and cell lysate preparation were as described above. Biotinylated antisense DNA oligonucleotide probes (100 pmol) and magnetic streptavidin beads (Thermo Fisher Scientific) were added to extracts. After incubation at room temperature for 4 h, beads were washed with NET-2 buffer and bound RNAs and associated proteins were subjected to RT–PCR and immunoblotting, respectively. For DNase treatment, washed beads were treated with DNase I (2 U/ml, Promega) and incubated at 37°C for 30 min followed by a wash with NET-2 buffer. For mass spectrometry (MS), samples were fractionated on SDS–polyacrylamide gels and stained with Coomassie blue. The bands of interest were excised, trypsinized and subjected to the nanoAcquity UPLC system (Waters) coupled with the Orbitrap Exploris™ 480 mass spectrometer (Thermo Fisher Scientific). This experiment was performed without repetition.

In brief, peptide mixtures were separated on a BEH C18 column (130 Å, 1.7 μm, 75 μm × 250 mm, Waters) using a gradient in 30 min from 5% to 35% solvent B (solvent B, 0.1% formic acid in acetonitrile; solvent A, 0.1% formic acid) at a flow rate of 300 nl/min. The mass spectrometer was operated in data-dependent acquisition mode. Full MS resolutions were set to 60 000 at *m/z* 200, and MS^2^ resolutions were set to 15 000. Isolation width was set at 1.3 *m/z*. Normalized collision energy was set at 30%. The raw files were searched against an *in silico* tryptic digest of the UniProt human proteome database using the Mascot search engine v.2.6.1 (Matrix Science). The search parameters included the mass tolerance of precursor peptide was set as 10 ppm and the tolerance for MS/MS fragments was 0.02 Da, cysteine carbamidomethylation as a fixed modification, variable oxidation of methionine and variable deamidation of asparagine or glutamine. Peptide spectrum matches were verified by a 1% FDR. We obtained 13 529 peptides with a SEQUEST score >20, which represented 3990 proteins in the MASCOT search (Supplementary Table S3). Among them, 688 proteins showed ≥5 protein member hits (Supplementary Table S4). The raw files of mass data have been deposited in the ProteomeXchange Consortium via the PRIDE (https://www.ebi.ac.uk/pride) partner repository with the dataset identifier PXD034470.

### Single-cell gel electrophoresis

This assay was carried out using the Comet Assay Kit (Abcam). Briefly, HeLa cells were transiently transfected with siRNAs or GapmeRs. Cells were harvested 48 h post-transfection and resuspended at 1 × 10^5^ cells/ml in PBS. Cells and comet agarose were mixed at 37°C at 1/10 ratio and 150 μl of the mixture was added onto the comet slide followed by incubation at 4°C for 15 min. The comet slide was immersed in the lysis buffer at 4°C for 1 h and subsequently in alkaline solution at 4°C for 30 min in the dark. Then the slide was subjected to electrophoresis in TBE running buffer at 20 V at 4°C for 30 min. After electrophoresis, the slide was immersed in H_2_O for 2 min followed by fixation with 70% ethanol for 5 min, and air dried. Cells were stained with Vista green DNA dye and images were acquired by a laser-scanning confocal microscope (LSM 780, Carl Zeiss). Data processing was performed using OpenComet plugin in ImageJ.

### NHEJ assays

To assess the NHEJ activity, two systems were adopted. For Cas9-mediated cleavage, the Cas9/sgHPRT-expressing vector and double-stranded DNA oligonucleotides Ins (25 pmol, Thermo Fisher Scientific) (34) together with siRNAs, GapmeRs or SMG6-expressing vectors were transfected into HeLa cells. Genomic DNAs were collected 48 h post-transfection and extracted by the PureLink™ Genomic DNA Mini Kit (Thermo Fisher Scientific). Total RNA was extracted with TRIzol reagent (Thermo Fisher Scientific) and then subjected to reverse transcription with SuperScript III (Thermo Fisher Scientific). For qPCR, the reactions containing 100 ng of genomic DNA or cDNAs, specific primers and PerfeCTa SYBR Green FastMix PCR Reagent (Quanta Biosciences) were performed in a LightCycler 480 Real-Time PCR System (Roche). For a GFP-based reporter assay, siRNAs, GapmeRs or SMG6-expressing vectors and the pSCE expression plasmid were transfected into HeLa GFP reporter cells (TopoGEN). Cells were harvested 72 h post-transfection. GFP-positive cells were detected by fluorescence-activated cell sorting using 1a 7-color LSR II Analytic Flow Cytometer (BD Biosciences).

### *In vitro* pull-down assay

Recombinant His-tagged Y14 fusion proteins (full-length and ΔC) were described previously (31,35). Recombinant His-tagged human Ku70/Ku80 heterodimer was purchased from Sino Biological. For *in vitro* pull-down assay, 5 μg of His-tagged proteins were incubated with 1 μg of total RNA extracted from HeLa cells and His•Bind Resin (Novagen) at 4°C for 2 h. After extensive washing, bound RNAs and proteins were analyzed by RT–PCR and immunoblotting, respectively.

### Immunoblotting

The procedure of immunoblotting was described previously (31). Antibodies used are listed in Supplementary Table S7.

### Laser microirradiation

U2OS cells were seeded in a chambered coverglass (Thermo Scientific) and transiently transfected with pcDNA-*HOTAIRM1*-6× MS2 (full-length, Δ5′, ΔE2 or Δ3′) and pcDNA-MCP-GFP or GapmeRs. Laser microirradiation was performed 48 h post-transfection using a laser-scanning confocal microscope (LSM 780, Carl Zeiss) and a 405 nm laser diode. After laser microirradiation, cells were fixed with 4% paraformaldehyde and permeabilized in 0.5% Triton X-100 in PBS. Immunofluorescence was performed by sequential incubation with primary and secondary antibodies. Nuclei were counterstained in Mounting Medium with 4′,6-diamidino-2-phenylindole (DAPI; Sigma). Samples were visualized using a laser-scanning confocal microscope (LSM 780, Carl Zeiss) coupled with an image analysis system.

For live cell imaging, U2OS cells that stably expressed GFP-fused Ku70, Ku80 or MDC1 (33) were transiently transfected with pLKO.1-sh*HOTAIRM1*. Laser microirradiation and time lapse imaging were carried out in a SP5 X inverted confocal microscope (Leica Microsystems) using laser diodes at 405 nm and 488 nm, respectively.

### Fluorescence *in situ* hybridization

*HOTAIRM1* was detected by fluorescence *in situ* hybridization (FISH) using bHM1-3 as probe (GENOMICS). After laser microirradiation, U2OS cells were fixed with 4% paraformaldehyde and permeabilized in 0.5% Triton X-100 in PBS. After washing with PBS, hybridization was performed in hybridization buffer containing 10% dextran sulfate, 2× SSC, 10% formamide, 2 mM ribonucleoside–vanadyl complex and bHM1-3 at 37°C for 24 h. After hybridization, cells were washed with PBS and incubated with anti-γH2AX, followed by Texas Red-conjugated anti-biotin and fluorescein isothiocyanate (FITC)-conjugated anti-mouse IgG. Nuclei were counterstained in Mounting Medium with DAPI (Sigma). Samples were visualized using a laser-scanning confocal microscope (Zeiss LSM 880 Airyscan Confocal microscope, Carl Zeiss) coupled with an image analysis system.

### Immunofluorescence

For immunofluorescence, U2OS cells after 10 Gy ionizing radiation (IR) were fixed with 4% paraformaldehyde and permeabilized in 0.5% Triton X-100 in PBS. Cells were incubated with antibodies against γH2AX, Upf1 or SMG6 followed by incubation with FITC-conjugated anti-mouse IgG (Cappel) or Alexa Fluor 568-conjugated anti-rabbit IgG (Thermo Fisher Scientific). Nuclei were counterstained in Mounting Medium with DAPI. Samples were visualized using a laser-scanning confocal microscope (LSM 780, Carl Zeiss) coupled with an image analysis system.

To detect the kinetics of foci formation, U2OS cells were irradiated and harvested at the indicated time points after IR. Cells were washed with PBS and pre-extracted with CSK buffer containing 0.3 mg/ml RNase A (42,43). After pre-extraction, cells were fixed with 2% paraformaldehyde (PFA) and permeabilized with 0.2% Triton X-100 in PBS. Cells were then sequentially incubated with primary antibodies against γH2AX or Ku70 and secondary antibodies. Samples were visualized using a laser-scanning confocal microscope (Zeiss LSM 880 Airyscan Confocal microscope, Carl Zeiss) coupled with an image analysis system.

### RT–qPCR and qPCR

Total RNA was extracted using TRIzol reagent (Thermo Fisher Scientific) followed by RNase-free DNase I (Promega) digestion and then subjected to reverse transcription with Random Hexamer Primer and Superscript III Reverse Transcriptase (Thermo Fisher Scientific). For real-time qPCR, the reactions were conducted in triplicate in a total volume of 20 μl, containing 100 ng of genomic DNA or cDNAs, specific forward and reverse primers (500 nM) and 10 μl of a SYBR Green FastMix PCR Reagent (Quanta Biosciences), and were performed in a LightCycler 480 Real-Time PCR System (Roche) following MIQE guidelines (44). Gene-specific primers were designed using Primer Blast (NIH). Differential expression analyses were performed with Student's *t*-test.

### Statistical analysis

Data are in general presented as means ± standard deviation (SD) from at least three independent experiments. Student's *t*-test was used to determine the statistical significance of two experimental groups. All statistical analyses were performed using GraphPad Prism 8.1.1 software (GraphPad). Statistical significance was indicated as n.s. not significant, *P <0.05, **P <0.01 and ***P <0.001.

## RESULTS

### Y14 is associated with non-coding RNAs

**Figure 1.**
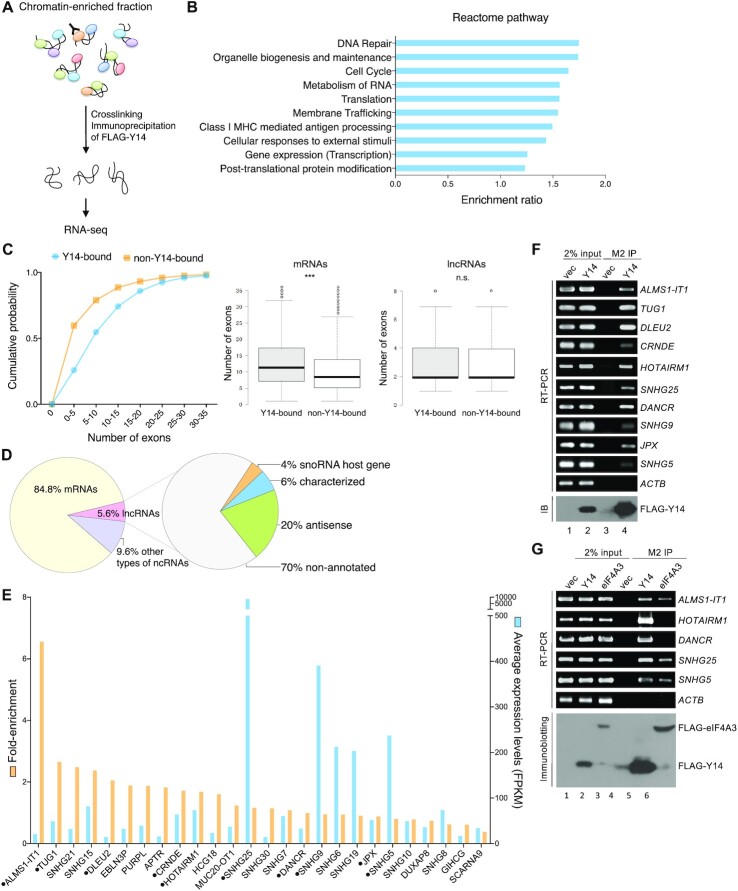
Identification of Y14-associated RNAs from chromatin-enriched fractions. (**A**) The diagram illustrates the procedure for identifying Y14-bound RNAs in the chromatin-enriched fraction of HEK293 cells that transiently expressed FLAG-Y14 through immunoprecipitation using anti-FLAG. (**B**) Reactome pathway analysis of proteins encoded by Y14-associated mRNAs. The bar graph shows the top 10 enriched pathways ranked by the enrichment ratio, i.e. the number of observed genes divided by the number of expected genes from each reactome pathway. (**C**) Graph showing the distribution of the number of exons per gene (Y14-bound RNAs versus non-Y14-bound RNAs). The average number of exons of Y14-associated mRNAs (middle panel; ***P<0.001) and lncRNAs (right panel; n.s., not significant) is shown. (**D**) Pie charts show the percentage of different classes of Y14-associated RNAs (mRNA, lncRNA, other types of ncRNA) (left) and lncRNAs (right). (**E**) Bar graph shows 26 annotated and high-abundance lncRNAs having an average FPKM value >15 in Y14-IP. Orange and blue represent fold enrichment and FPKM value, respectively. A dot indicates lncRNAs selected for experimental verification. (**F**) HEK293 cells were transiently transfected with empty (vec) or FLAG-Y14-expressing vector. Cell lysates were subjected to immunoprecipitation (IP) using anti-FLAG, followed by RT–PCR using primers specific for the indicated lncRNAs or β-actin (*ACTB*, control). Immunoblotting was performed using anti-FLAG. (**G**) HEK293 cells were transiently transfected with empty (vec), or FLAG-Y14- or FLAG-eIF4A3-expressing vector. IP, RT–PCR and immunoblotting were as in (F).

We previously established that RNA mediates the interaction between Y14 and Ku70/80 (31). Y14 is more abundant than eIF4A3, another EJC factor, in the chromatin-enriched fraction, and further accumulates on chromatin with Ku70/80 after DNA damage (31). To identify putative Y14-associated RNAs, we overexpressed FLAG-Y14 in HEK293 cells and performed RIP using anti-FLAG with the chromatin-enriched fraction. Co-precipitated RNAs (Y14-IP) were subjected to high-throughput RNA sequencing using an Illumina HiSeq platform (Figure 1A). RIP coupled with RNA sequencing was performed in duplicate. Meanwhile, a controlled RIP-seq was performed in parallel using the chromatin fraction of mock-transfected HEK293 cells (vec-IP). Data analysis was performed using the NOISeq R-package, revealing 27 903 transcripts [FPKM (fragments per kilobase of transcript per million reads) values >0] in at least one Y14-IP sample (Supplementary Table S1, Total). Among them, 6164 transcripts (5229 mRNAs and 935 non-mRNAs, including lncRNAs, pseudogenes and nuclear/nucleolar small RNAs) with an averaged FPKM value >10 in Y14-IP and zero counts in the control (vec-IP) were referred to as Y14-associated transcripts (Supplementary Table S1). Reactome pathway enrichment analysis revealed that proteins encoded by Y14 target mRNAs have a role in DNA repair, cell cycle and RNA metabolism (Figure 1B; Supplementary Table S2), consistent with previous findings that Y14 regulates the expression or splicing of DNA repair- or cell cycle-related factors (29,31). Moreover, Y14-associated RNAs had a significantly greater number of exons compared with non-associated RNAs, supporting the role of Y14 as an EJC component or suggesting the association of Y14 with primary transcripts during splicing (Figure 1C, left). Using the lncRNA database LNCipedia (41), we identified 349 (∼5.6% of the total) lncRNAs that were associated with Y14 (Supplementary Table S1, LNCipedia). Unlike Y14 target mRNAs, there was no difference in exon number between Y14-associated and non-associated lncRNAs (Figure 1C, middle for mRNAs and right for lncRNAs). Among these identified lncRNAs, ∼30% have been annotated, including small nucleolar RNA host genes (*SNHG*s) and previously characterized lncRNAs and antisense RNAs, whereas ∼70% were of unknown identity (Figure 1D).

To experimentally verify Y14-associated lncRNAs, we selected three *SNHG* RNAs and seven lncRNAs from a set of 26 annotated and high-abundance candidates in Y14-IP (FPKM values >15; fold enrichment ranging from 0.4 to 6.57) (Figure 1E; Supplementary Table S1, lncRNA). Immunoprecipitation followed by RT–PCR showed that FLAG-Y14 associated with all the selected lncRNAs but not β-actin mRNA (control), indicating the specificity of our affinity selection (Figure 1F). Furthermore, we observed that both FLAG-tagged Y14 and eIF4A3 interacted with two *SNHG* RNAs and *ALMS1-IT1*, whereas only Y14 co-precipitated *HOTAIRM1* and *DANCR* (Figure 1G). The association of both Y14 and eIF4A3 with *SNHG*s echoed a previous finding that the EJC participates in their processing and/or NMD (45).

### *HOTAIRM1* mediates the interaction between Y14 and Ku70/80

**Figure 2.**
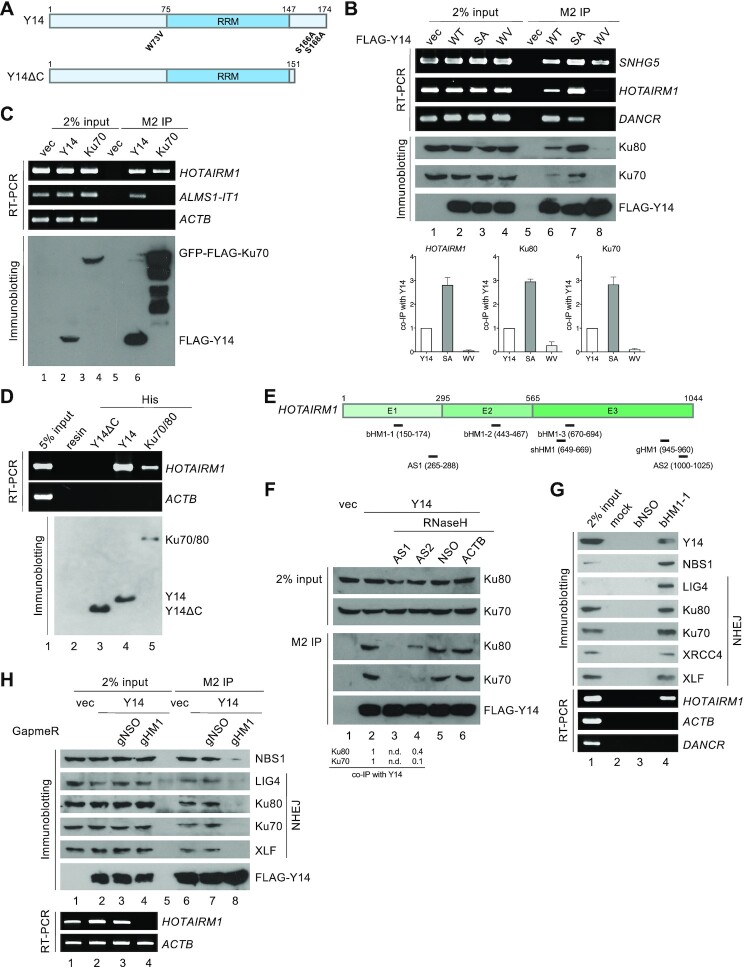
*HOTAIRM1* is associated with the NHEJ complex. (**A**) Domains of Y14 (RRM, RNA recognition motif) and its mutants (WV and SA) and C-terminal truncated version. (**B**) HEK293 cells were transfected with empty (–) or FLAG-Y14-expressing vector (WT, SA or WV mutant; WT represents the wild type), followed by immunoprecipitation (IP), and RT–PCR or immunoblotting. Bottom: bar graphs show the relative co-precipitation efficiency of *HOTAIRM1*, Ku80 and Ku70 with each Y14 version (mean ± SD). *n* (the number of experimental repeats) = 4. (**C**) HEK293 cells were transiently transfected with empty (vec), or FLAG-Y14- or GFP–FLAG-Ku70-expressing vector, followed by immunoprecipitation along with RT–PCR or immunoblotting using anti-FLAG. (**D**) Recombinant His-tagged Y14, Y14ΔC or Ku70/80 was incubated with total HeLa cell RNAs, followed by pull-down using nickel resin. Bound RNAs were detected by RT–PCR. Bottom panel shows SDS–PAGE of recombinant proteins. (**E**) Oligonucleotides (AS, antisense; bHM1, biotinylated; gHM1, GapmeR) and short hairpin RNA (shHM1) complementary to *HOTAIRM1*. E indicates exon. (**F**) HEK293 cells were transfected with empty vector (vec) or FLAG-Y14 vector. Anti-FLAG immunoprecipitates were mock treated (lane 2) or incubated with antisense oligonucleotides in the presence of RNase H (lanes 3–6). Immunoblotting was performed using antibodies against the indicated proteins. NSO and ACTB represent non-specific and β-actin-targeting antisense oligonucleotides, respectively. Bottom: the numbers indicate the relative level of Ku70 and Ku80 in Y14 co-precipitates after destruction of *HOTAIRM1*. *n* = 3; n.d., not detectable. (**G**) HEK293 cell lysates were mock incubated (lane 2) or incubated with biotinylated oligonucleotides (lanes 3 and 4), followed by pull-down using strepatavidin agarose and immunoblotting or RT–PCR. bNSO: biotinylated non-specific oligonucleotide. (**H**) HeLa cells were transfected with empty vector (vec) or FLAG-Y14 vector or together with the indicated GapmeR. Anti-FLAG immunoprecipitates were subjected to immunoblotting and RT–PCR. gNSO: non-specific GapmeR. gHM1 disrupted the interaction of Y14 with NBS1 and NHEJ factors by >90%.

To further pinpoint which lncRNA may have a role in the Y14-mediated DNA repair pathway, we evaluated the interaction of wild-type and mutant Y14 (Figure 2A) with three lncRNAs and Ku70/80. Our previous reports have identified phosphorylation sites and RNA-binding mutations of Y14 (35,45). Therefore, we attempted to evaluate the affinity of the non-phosphorylatable Y14-SA (S166,168A) and RNA-binding mutant Y14-WV (W73V) in lncRNAs and NHEJ factors. As compared with wild-type Y14, Y14-SA was more abundant in the chromatin fraction (Supplementary Figure S1A) and co-precipitated with Ku70/80 to a greater extent, whereas Y14-WV failed to interact with Ku70/80 (Figure 2B). Among three lncRNAs examined (Figure 1G), only *HOTAIRM1* behaved similarly to Ku, showing a greater affinity for Y14-SA and not interacting with Y14-WV (Figure 2B), suggesting that *HOTAIRM1* is a candidate that mediates the interaction between Y14 and Ku. Moreover, the observation that Y14-WV interacted with neither *HOTAIRM1* nor Ku70/80 supported the idea that the Y14–Ku70/80 interaction is RNA dependent. Since Y14-WV is able to form the EJC (46), its association with *SNHG5* was probably via other components (such as eIF4A3) of the EJC. Moreover, *HOTAIRM1* was co-precipitated with FLAG-tagged GFP–Ku70 (Figure 2C) and GFP–Ku80 (Supplementary Figure S1B). Deletion of the central domain of Ku80 disrupted its interaction with Ku70 and *HOTAIRM1* (Supplementary Figure S1B), suggesting that the heterodimerization of Ku70/80 is important for *HOTAIRM1* interaction. Moreover, recombinant His-tagged Y14 and Ku70/80 could pull down *HOTAIRM1* from HeLa cell total RNA (Figure 2D, lanes 4 and 5) and directly bind to *HOTAIRM1* in an electrophoresis mobility shift assay (EMSA; Supplementary Figure S1C). However, His-tagged Y14-ΔC lacking the last 23 amino acids could not (Figure 2D, lane 3), indicating that the C-terminal arginine-rich domain of Y14 is essential for its interaction with *HOTAIRM1*. Next, to examine whether *HOTAIRM1* indeed mediates the interaction between Y14 and Ku70/80, we performed immunoprecipitation of FLAG-Y14 in the HEK293 cell lysate. RNase H-mediated digestion of *HOTAIRM1* in the presence of its antisense oligonucleotide dissociated Ku70/80 from Y14 immunoprecipitates (Figure 2E, F; Supplementary Figures S1D, AS1 and AS2). Non-specific or antisense ACTB oligonucleotide (Figure 2F) or mock treatment (without RNase H) (Supplementary Figure S1E) had no effect. This result supported a scaffold role for *HOTAIRM1*.

### *HOTAIRM1* is essential for the association of Y14 with the NHEJ complex

To investigate whether *HOTAIRM1* associates with the entire NHEJ, we performed RNA affinity selection using a biotinylated oligonucleotide complementary to *HOTAIRM1* with HEK293 cell lysates (Figure 2E; Supplementary Figure S1D, bHM1-1). bHM1-1 specifically pulled down *HOTAIRM1* as well as Y14, the MRN component NBS1 and all NHEJ factors, i.e. DNA ligase 4 (LIG4), Ku70/80, XRCC4 and XLF (Figure 2G; see Supplementary Figure S1F for the two additional biotinylated oligonucleotides). Affinity selection of an irrelevant lncRNA, *Gas5*, did not pull down Y14 or any DNA repair factors examined (Supplementary Figure S1G). Moreover, DNase treatment had no significant effect on the integrity of the complex formed by *HOTAIRM1*, Y14 and NHEJ factors (Supplementary Figure S1H).

Next, to evaluate whether *HOTAIRM1* mediates the interaction between Y14 and the NHEJ factors *in vivo*, we transfected HEK293 cells with a *HOTAIRM1*-targeting GapmeR (Figure 2E; Supplementary Figure S1C, gHM1). Upon transfection with gHM1, *HOTAIRM1* was almost undetectable (Figure 2H, RT–PCR, lane 4). Under this condition, FLAG-Y14 no longer co-precipitated any NHEJ factors (Figure 2H, lane 8). A non-specific oligonucleotide (gNSO) had no effect on the Y14–NHEJ complex (lane 7). Destruction of *HOTAIRM1* also disrupted the association of Ku70 with other NHEJ factors to different extents (Supplementary Figure S1I). Therefore, *HOTAIRM1* may not only mediate the interaction between Y14 and the NHEJ factors, but also contributes to the formation or stabilization of the NHEJ complex. Finally, we observed that the interaction between *HOTAIRM1* and Y14 and Ku was not induced by IR but was sensitive to ATM (ataxia telangiectasia mutated) inhibition (Supplementary Figure S1J). This was in line with our previous finding that the Y14–Ku70/80 interaction is independent of DNA damage but requires ATM activity (31).

### *HOTAIRM1* is essential for genome integrity and accumulates at DNA damage sites

**Figure 3.**
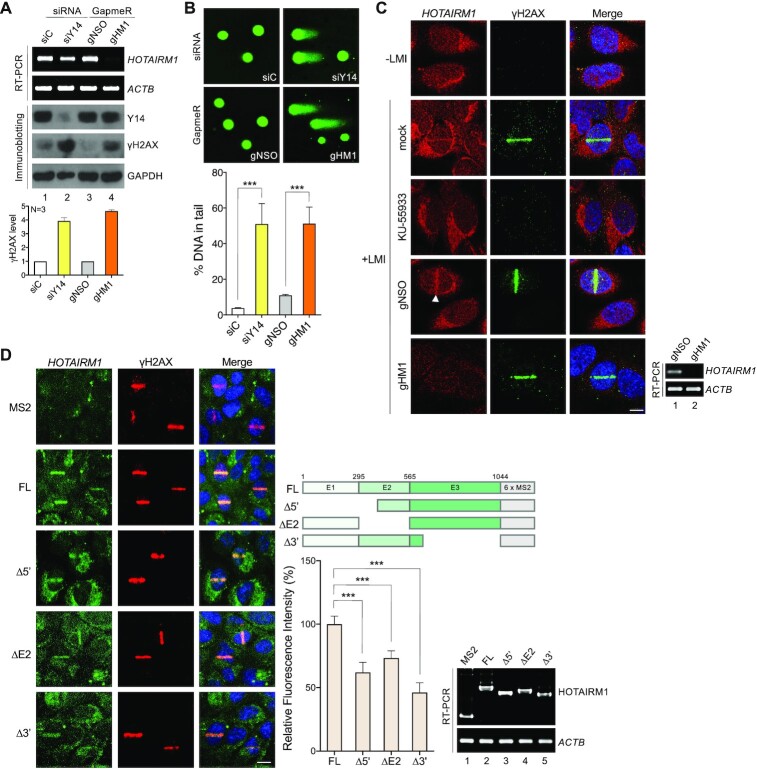
*HOTAIRM1* accumulates at DNA damage sites and contributes to genome integrity. (**A**) HeLa cells were transfected with the indicated siRNA or GapmeR. RT–PCR and immunoblotting were performed 48 h post-transfection. Bottom: the bar graph shows fold increase of γH2AX after knockdown of Y14 or *HOTAIRM1* (mean ± SD; *n* = 3). (**B**) HeLa cells were transfected as in (A), followed by the comet assay. Bar graph shows the percentage of DNA in the comet tail (mean ± SD; ***P<0.001 for a two-tailed test); for each sample, 46 or 57 cells were quantified. (**C**) U2OS cells were untransfected but were mock or Ku-55933 treated as indicated (upper three rows) or transfected with GapmeRs (lower two rows). Except for a set of untransfected cells (–LMI, the top row), laser microirradiation (405 nm; +LMI) was performed. Cells were subsequently immediately fixed for *in situ* hybridization using bHM1-3 as the probe and subsequently subjected to immunofluorescence microscopy using anti-γH2AX. Arrowhead, LMI track. Scale bar, 10 μm. (**D**) Diagram shows *HOTAIRM1–*MS2 chimeric RNA and truncations. U2OS cells were transfected with the control vector (MS2 only) or a *HOTAIRM1*–MS2-expressing vector and the GFP–MCP-expressing vector (GFP signals represent *HOTAIRM1*), followed by indirect immunofluorescence microscopy using anti-γH2AX. Bar graph shows the relative efficiency (GFP/γH2AX intensities) of truncated *HOTAIRM1* fragments in localization to the DSB sites; the full-length *HOTAIRM1* was set to 100% (mean ± SD; ***P<0.001 for a two-tailed test); for each sample, six or 11 cells were quantified. Scale bar, 20 μm. RT–PCR shows the expression of MS2 and full-length and truncated MS2–HOTAIRM1.

We have previously reported that Y14 depletion causes cell cycle arrest at the G_2_/M phase and increases the sub-G_1_ population and the radiosensitivity of HeLa cells (30). Knockdown of *HOTAIRM1* in HeLa cells by gHM1 also resulted in the above phenotypes, suggesting that *HOTAIRM1* depletion sensitizes cells to DNA damage (Supplementary Figure S2). Consistently, depletion of *HOTAIRM1* increased the level of the DSB marker γH2AX in HeLa cells (Figure 3A, lane 4), as also observed for Y14 depletion (lane 2) and Ku80 depletion (Supplementary Figure S3A). Single-cell gel electrophoresis (the comet assay) revealed that gHM1-mediated depletion of *HOTAIRM1* significantly increased the tail of cells, which reflects DNA damage (Figure 3B). All these results supported a role for *HOTAIRM1* in the maintenance of genome integrity.

Next, we examined whether endogenous *HOTAIRM1* is located at DNA damage sites. Use of bHM1-3 as a probe for FISH revealed that *HOTAIRM1* was distributed primarily in the cytoplasm but also in the nucleus of U2OS cells, as previously reported (47) (Figure 3C, untransfected). Laser microirradiation induced *HOTAIRM1* accumulation at DNA damage tracks, as indicated by γH2AX (Figure 3C, arrowhead). No FISH signal was detected in *HOTAIRM1*-depleted cells, indicating the specificity of the FISH probe (Figure 3C, gHM1). ATM inhibition abolished the signals of both γH2AX and *HOTAIRM1* (Figure 3C, KU-55933). Minor or no effect was observed upon inhibition with DNA-PK (Nu7441) or MRN (Mirin) (Supplementary Figure S3B). Treatment of cells with IR increased the nuclear/cytoplasmic ratio of *HOTAIRM1* in U2OS cells (Supplementary Figure S3C). IR or DSB-inducing agents (phleomycin or zeocin) resulted in *HOTAIRM1* accumulation on chromatin (Supplementary Figure S3D). To confirm the relocation of *HOTAIRM1* to DNA damage sites, we tagged *HOTAIRM1* with six repeats of the RNA stem–loop of bacteriophage MS2, which can be recognized by the MS2 capsid protein (MCP). U2OS cells were co-transfected with a vector expressing *HOTAIRM1*–MS2 and GFP–MCP. Endogenous *HOTAIRM1* was estimated to be 60–70 molecules per U2OS cell (Supplementary Figure S3E). Transiently expressed *HOTAIRM1–*MS2 and its truncated fragments were 3-5-fold more abundant than the endogenous form (Supplementary Figure S3F). Indirect immunofluorescence revealed that *HOTAIRM1–*MS2, but not MS2 or β-globin–MS2, localized to laser microirradiation-induced DNA damage tracks (Figure 3D; Supplementary Figure S3G). Moreover, using a proximity-ligation assay, we observed that phleomycin induced the physical interaction between γH2AX and endogenous *HOTAIRM1* but not *Gas5* (Supplementary Figure S3H). These results together indicated that *HOTAIRM1* is specifically associated with DNA damage sites.

During the course of this study, we found that *HOTAIRM1* existed in two isoforms, i.e. full-length and the exon 2-skipped isoform (ΔE2). Their expression ratio differed between cell lines; the latter was somewhat dominant in U2OS cells (Supplementary Figure S3I). Nevertheless, both could associate with Y14 and Ku70 (Supplementary Figure S3J). MS2-tagged ΔE2 accumulated at DNA lesions after laser microirradiation, albeit less efficiently than the full-length isoform (Figure 3D, bar graph). We also generated the 5′- or 3′-truncated versions. Among all truncated forms, the 3′ 431 nucleotide truncation more severely affected *HOTAIRM1* localization (Figure 3D, Δ3′). The question of how each segment associates with DNA repair factors warrants further investigation.

### *HOTAIRM1* modulates the recruitment and retention of DNA repair factors at DNA damage sites

**Figure 4.**
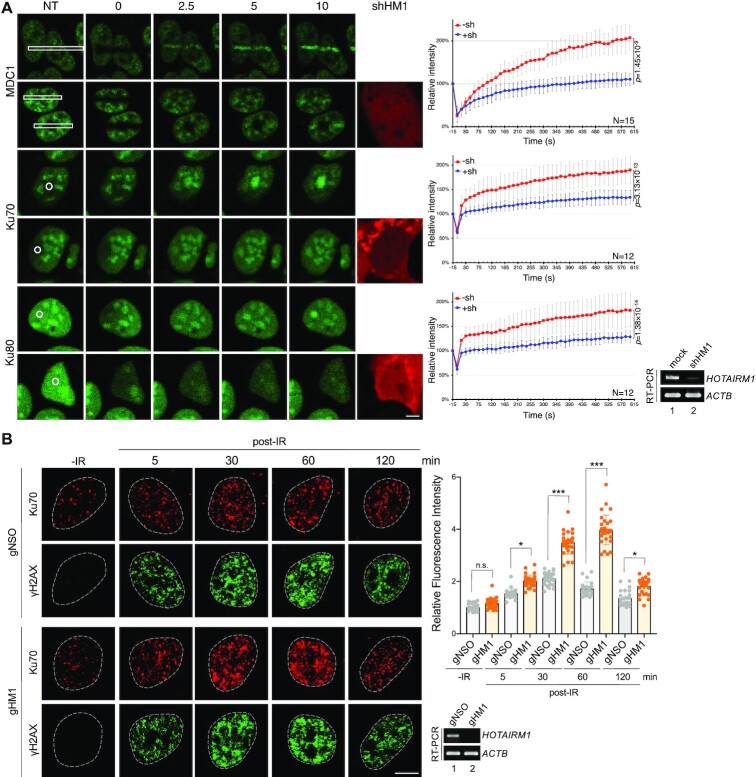
*HOTAIRM1* is required for the recruitment of DNA repair factors to DSB sites and regulates Ku foci dynamics. (**A**) U2OS cells that stably expressed the GFP fusion with MDC1, Ku70 or Ku80 were mock transfected or transfected with the shHORAITM1 (shHM1)–mCherry-expressing vector. Cells were subjected to laser microirradiation followed by live-cell imaging using confocal microscopy. Representative confocal images show accumulation of GFP fusion proteins at sites (white-outlined rectangles or circles) of laser microirradiation at the indicated time points. NT (non-treated) indicates samples before microirradiation. mCherry represents sh*HOTAIRM1*-expressing cells. Graphs to the right show fluorescence intensities of GFP fusion proteins at the irradiated region. Intensity was quantified periodically up to 10 min, normalized, and is presented as the mean and SD (*P*-values as indicated) for 12 or 15 cells in each experiment. The RT–PCR data indicate the efficiency of *HOTAIRM1* knockdown. Scale bar, 10 μm. (**B**) U2OS cells were transfected with gNSO or gHM1. Cells were not irradiated (–IR) or exposed to 10 Gy of IR and harvested at the indicated time points post-IR. Cells were treated with Triton X-100 and RNase A according to Britton *et al.* (43). Indirect immunofluorescence microscopy was performed using anti-γH2AX and anti-Ku70. Dot graph shows relative fluorescence intensity of Ku70; for each sample, 21–29 cells were measured (–IR was set to 1; mean ± SD; *P*-values for a two-tailed test, *<0.05, ***<0.001, n.s. not significant). Scale bar, 10 μm. RT–PCR in both panels shows *HOTAIRM1* knockdown efficiency.

Next, we took advantage of live-cell imaging of U2OS cells that stably expressed a GFP fusion with MDC1 or Ku70/80 (33) to evaluate the effect of *HOTAIRM1* on localization of repair factors to laser-induced DNA damage sites. These U2OS cells were transiently transfected with a vector expressing a *HOTAIRM1*-targeting short hairpin RNA (shHM1) (Figure 2E; Supplementary Figure S1C) and red fluorescent protein (RFP), which served as a marker of transfected cells. shHM1 down-regulated *HOTAIRM1* by up to 70% (Figure 4A, RT–PCR). Laser microirradiation caused a gradual accumulation of GFP-tagged DNA damage repair factors (MDC1, Ku70 and Ku80) at DNA damage sites in mock-transfected cells, and this accumulation was considerably reduced in shHM1-expressing (RFP-positive) cells (Figure 4A, fluorescence live-cell imaging and line graphs). This result indicated that *HOTAIRM1* is required for the recruitment of DNA repair factors. Depletion of Y14 leads to Ku accumulation on chromatin after DNA damage (31). Therefore, we examined whether *HOTAIRM1* depletion affects the IR-induced formation of Ku70 foci. Use of an RNase A-based extraction method revealed that IR transiently increased the signal of Ku70 foci in U2OS cells (Figure 4B, gNSO for 5 and 30 min), as previously reported (42). The Ku70 signal markedly increased from 5 to 60 min after IR treatment and returned to baseline at 2 h in *HOTAIRM1*-depleted cells (Figure 4B, gHM1). The kinetics of γH2AX foci formation did not significantly differ between control and *HOTAIRM1*-depleted cells (Figure 4B; Supplementary Figure S4A, γH2AX). This result was reminiscent of inhibition of Ku ubiquitination during DNA damage repair (42). Therefore, *HOTAIRM1* depletion may cause Ku retention or impair Ku removal from DNA repair sites. Together, these results suggested that *HOTAIRM1* promotes efficient loading of the repair factors to DSBs and is also required for their dissociation from DSBs.

### *HOTAIRM1* participates in NHEJ-mediated DSB repair

**Figure 5.**
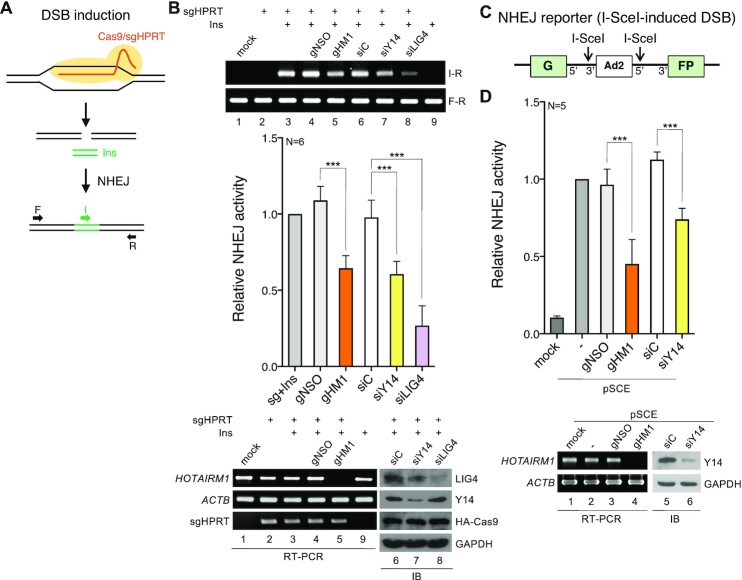
*HOTAIRM1* participates in DSB repair. (**A**) Experimental design for quantitative measurement of NHEJ-mediated repair of DSBs. Transfection of HeLa cells with the Cas9/sgRNA (sgHPRT) vector induces DSBs. Incorporation of the double-stranded oligonucleotide ‘Ins’ into the DSB sites evaluated by PCR and qPCR represented DNA repair efficiency. (**B**) HeLa cells were mock transfected (lane 1) or transfected for 48 h with one or more of the following reagents: the Cas9/sgHPRT plasmid, Ins and GapmeR (lanes 4 and 5) or siRNA (lanes 6–8), as indicated. Genomic DNA was recovered for PCR using the primer set I/R or F/R. Bar graph shows qPCR of lanes 3–8 (sg + Ins was set to 1; mean ± SD; *n* = 6; ***P<0.001 for a two-tailed test). Immunoblotting and RT–PCR indicate the knockdown efficiency of *HOTAIRM1*, Y14 and LIG4 and transfection efficiency of Cas9/sgHPRT; lanes are numbered to correspond with the PCR analysis (upper panel). (**C**) HeLa NHEJ reporter cells in which the chromosomally integrated GFP gene is disrupted by an intron and flanked by I-SceI sites. I-SceI-induced cleavage mimics a DSB. GFP expression was restored after the cleavage repaired through NHEJ. (**D**) HeLa NHEJ reporter cells were mock transfected or transfected with pSCE (the I-SceI expression vector) and GapmeR or siRNA as indicated. The number of GFP-positive cells was counted 48 h post-transfection. Bar graph represents relative repair efficiency; samples without GapmeR/siRNA transfection were set to 1 (mean ± SD; *n* = 5; ***P<0.001 for a two-tailed test). Immunoblotting and RT–PCR show knockdown efficiency.

Next, we adopted two assay systems to evaluate whether *HOTAIRM1* is essential for DSB repair. First, using CRISPR/Cas9-mediated DNA cleavage at a specific gene, namely *HPRT*, we analyzed DNA repair in the presence of a blunt-ended double-stranded oligonucleotide Ins (34) (Figure 5A). Insertion of Ins into the cleavage site, indicating DNA repair (34,48), was evaluated by PCR using primers complementary to Ins (primer I) and the region downstream of the cleavage site (primer R). The PCR product I-R was detectable upon transfection of cells with both the Cas9/sgHPRT vector and Ins (Figure 5B, upper, lane 3) but not detected by transfection with either alone (lanes 2 and 9). qPCR revealed that knockdown of Y14 or LIG4 using an siRNA reduced the efficiency of DNA repair by ∼50% and 70%, respectively, and *HOTAIRM1* depletion impaired DNA repair by ∼40% (Figure 5B, bar graph). To confirm the role of *HOTAIRM1* in NHEJ, we additionally used HeLa NHEJ reporter cells for analysis (49). The chromosomally integrated GFP reporter gene was disrupted by an intron, within which an inserted exon (Ad2) was flanked by two I-SceI sites (Figure 5C). Transfection of cells with the I-SceI expression vector (pSCE) induced cleavage, which mimicked a DSB. Upon DNA repair, the GFP reporter gene was expressed, producing the transcript, within which Ad2 had been removed by splicing. In general, ∼5% GFP-positive cells were observed in pSCE-transfected cells. Knockdown of Y14 or *HOTAIRM1* reduced the number of GFP-positive cells by 36% and 53%, respectively (Figure 5D, bar graph). To analyze whether *HOTAIRM1* has any role in HR, we took advantage of Cas9/sgHPRT-induced cleavage and template-mediated repair (34). For this HR assay, the Cas9/sgHPRT vector was co-transfected with a single-stranded DNA template containing the Ins sequence flanked by the sequence complementary to *HPRT* followed by qPCR using the primers I and R as above (34). Consistent with the previous report (34), the MRN inhibitor mirin reduced HR efficiency by 55% in HeLa cells. However, depletion of *HOTAIRM1* or Y14 had no significant effect (Supplementary Figure S5). These results suggested a role for *HOTAIRM1* in DSB repair probably via the NHEJ pathway.

### *HOTAIRM1* associates with DNA damage repair factors and RNA processing factors

**Figure 6.**
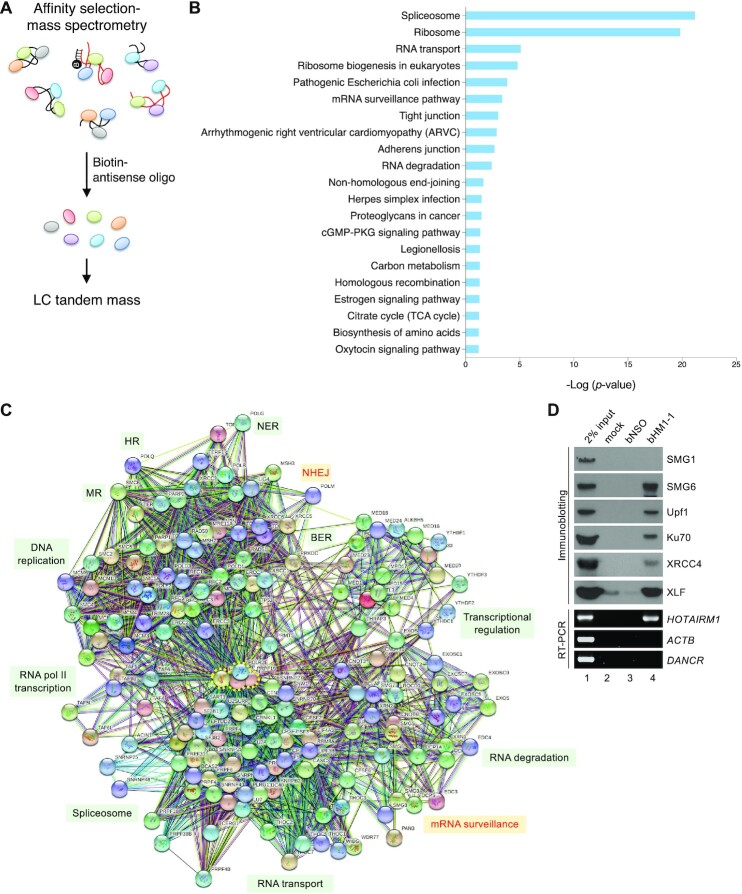
*HOTAIRM1* is associated with the mRNA surveillance factors. (**A**) Procedure for identification of *HOTAIRM1*-interacting proteins. HEK293 cell lysates were incubated with three bHM1 oligonucleotides followed by affinity selection using streptavidin. Selected proteins were analyzed by MS. (**B**) KEGG pathway enrichment analysis of the 688 *HOTAIRM1* partners that were identified. Bar graph shows the top enriched Gene Ontology terms (*P*-value <0.05) from the KEGG Pathway Database. (**C**) STRING analysis of the 166 *HOTAIRM1*-interacting proteins (Supplementary Table S6) that function in gene expression, DNA replication or DNA repair. The diagram shows protein–protein interaction (PPI) networks for these proteins (PPI enrichment *P*-value <1.0e-16). NHEJ and mRNA surveillance are highlighted in red. RNA polymerase II (RNA pol II) subunits and the PRP19–CDCL5 complex are enclosed by a dotted yellow line and represent hub proteins. BER, base excision repair; MR, mismatch repair; NER, nucleotide excision repair. (**D**) Affinity selection of *HOTAIRM1* was as in (A). RT–PCR and immunoblotting were performed to detect *HOTAIRM1* and its interacting partners.

To gain further insight into *HOTAIRM1*-mediated DNA repair, we affinity-selected *HOTAIRM1* in the lysates of UV-cross-linked HEK293 cells using three bHM1 oligonucleotides (Figure 6A). Co-purified proteins were gel-fractionated and analyzed by liquid chromatography–tandem MS. Among the 3990 proteins that were identified (Supplementary Table S3), 688 proteins with ≥5 protein member hits in the MASCOT search results were subjected to pathway enrichment analysis with the Kyoto Encyclopedia of Genes and Genomes (KEGG) using DAVID software (david.ncifcrf.gov) (Supplementary Table S5). *HOTAIRM1*-associated proteins included spliceosomal and ribosomal factors, which were at the top of the ranked list (respective *P*-values, 6.35E-22 and 1.44E-20), and various RNA processing and DNA damage repair factors (Figure 6B). The potential association of *HOTAIRM1* with ribosomal proteins was in line with its presence in the 40S ribosomal subunit and 80S mono-ribosome (Supplementary Figure S6A), but the significance of this observation warrants future investigation. Notably, some of the mRNA processing and surveillance factors identified have been implicated in DNA damage repair, such as the RNA exosome component EXOSC10/Rrp6 (50,51). The most highly represented DNA damage repair pathways were NHEJ and HR (respective *P*-values, 0.022 and 0.049), supporting a role for *HOTAIRM1* in DSB repair. Since MS analysis was performed without biological replication, any identified factors need to be verified by additional methods.

We arbitrarily selected 166 proteins that form functional complexes involved in transcription, RNA processing, DNA replication or repair, or genome stability for analysis of protein–protein interaction networks using STRING (string-db.org) (Supplementary Table S5). The results underscored *HOTAIRM1-*mediated connection between transcription, RNA processing and various DNA repair pathways (Figure 6C). Moreover, identification of PARP1, the MRN complex and four NHEJ factors (Ku80, Ku70, DNA-PK and LIG4) supported the role of *HOTAIRM1* in the NHEJ pathway. Although the above result argued against the role of *HOTAIRM1* in HR, we cannot exclude the possibility that *HOTAIRM1* participates in the repair of any other types of DNA damage (Figure 6C; Supplementary Table S6). Notably, we identified a set of NMD factors, namely Upf1, Upf3B, SMG5, SMG6, SMG7, SMG8 and SMG9. Although NMD factors function in cytoplasmic mRNA surveillance, some of them, such as Upf1 and SMG6, have been implicated in genome/telomere integrity (52–54). Moreover, a recent report has revealed a role for Upf1 in HR-mediated DSB repair (55). Affinity selection and immunoblotting confirmed the association of these two NMD factors with *HOTAIRM1* (Figure 6D) but not *Gas5* (Supplementary Figure S6B). Notably, the unidentified factor SMG1 was not detected in the *HOTAIRM1* pull-down (Figure 6D). Association of Upf1 and SMG6 with *HOTAIRM1* was DNA damage (IR) independent (Supplementary Figure S6C) but was sensitive to ATM inhibition (Supplementary Figure S1I). Moreover, RNase treatment revealed that Upf1 but not SMG6 directly interacted with Y14, suggesting that Y14 recruits Upf1 to *HOTAIRM1* (Supplementary Figure S6D). SMG6 binds to Upf1 (36). Together, by affinity selection of *HOTAIRM1*, we found that its ribonucleoprotein complex contained a set of NMD factors.

### The mRNA surveillance factors Upf1 and SMG6 participate in DSB repair

**Figure 7.**
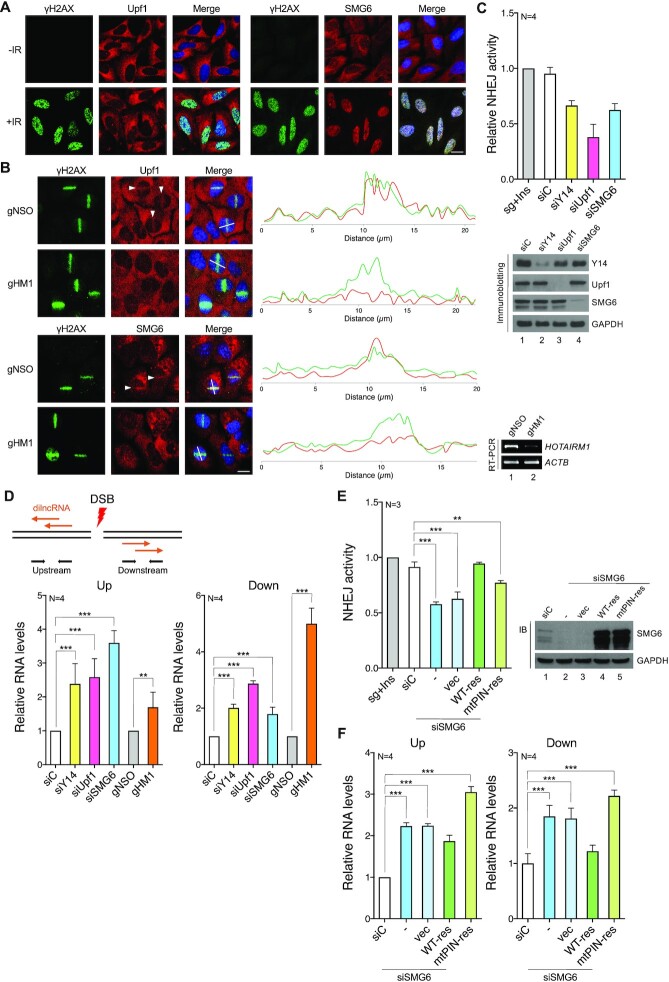
mRNA surveillance factors are involved in DSB repair. (**A**) U2OS cells were mock irradiated (–IR) or irradiated with 10 Gy (+IR) followed by immunofluorescence microscopy using antibodies against the indicated proteins. Scale bar, 20 μm. (**B**) U2OS cells were transfected with gNSO or gHM1. Laser microirradiation was performed, followed by immunofluorescence microscopy using antibodies against the indicated proteins. Arrowheads indicate laser-irradiated cells. Fluorescence intensities along a white line across a nucleus were measured in arbitrary units. Line-scan profiles of fluorescence intensity are shown to the right. RT–PCR shows *HOTAIRM1* knockdown efficiency. Scale bar, 20 μm. (**C**) The DSB repair assay was performed as in Figure 5B. HeLa cells were transfected with the Cas9/sgHPRT vector, Ins and siRNA as indicated. Genomic DNA was collected at 48 h post-transfection and subjected to qPCR. Bar graph is shown as in Figure 5B (mean ± SD; *n* = 4; ***P<0.001 for a two-tailed test). (**D**) Schematic drawing of Cas9/sgHPRT-generated DSBs and production of dilncRNAs in the DSB-flanking regions. HeLa cells were transfected with the Cas9/sgHPRT vector, Ins, siRNA or GapmeR as indicated. RT–qPCR was performed using the primers indicated in the diagram. Bar graphs show relative levels of dilncRNAs (mean ± SD; *n* = 4; ***P<0.001 for a two-tailed test). (**E**) HeLa cells were transfected with the Cas9/sgHPRT vector, Ins, siC or siSMG6 together with the empty vector (vec) or the siRNA-resistant wild-type or mutant SMG6 expression vector (WT-res or mtPIN-res). qPCR and immunoblotting were performed as in (C) (mean ± SD; *n* = 3; *P*-values for a two-tailed test, **<0.01, ***<0.001). (**F**) HeLa cells were transfected as in (E). RT–qPCR was performed as in (D) (mean ± SD; *n* = 4; ***P<0.001 for a two-tailed test).

Next, we investigated whether Upf1 and SMG6 participate in DNA damage repair. Both Upf1 and SMG6 were found to reside predominantly in the cytoplasm of U2OS cells (Figure 7A, –IR). To our surprise, IR induced drastic nuclear translocation of SMG6, although Upf1 was still largely retained in the cytoplasm (Figure 7A, +IR). IR also induced the accumulation of Upf1 and SMG6 on chromatin (Supplementary Figure S7A). Strikingly, these two NMD factors localized to laser-induced DNA damage tracks (Figure 7B; transfection with gNSO), suggesting their potential involvement in DNA damage repair. It is noteworthy that depletion of *HOTAIRM1* abolished the laser-induced localization of Upf1 and SMG6 at DNA damage sites (Figure 7B; gHM1), indicating that *HOTAIRM1* escorts mRNA surveillance factors to DNA lesions. This result reinforced a specific role for *HOTAIRM1* in DSB repair. Next, using Cas9/sgHPRT-induced DNA cleavage, we assessed whether these two factors have a role in DNA repair. Depletion of Upf1 or SMG6 by siRNA reduced DNA repair efficiency by ∼60% and 35%, respectively (Figure 7C). A similar experiment was performed in HeLa NHEJ reporter cells. The result further supported the role of Upf1/SMG6 in efficient DSB repair (Supplementary Figure S7B). Furthermore, we knocked down two components in the *HOTAIRM1* complex in the Cas9/sgHPRT-based NHEJ assay. As compared with single depletion, dual depletion further suppressed the NHEJ activity by 30–60% (Supplementary Figure S7C), suggesting that these factors not only may function coordinately but also have independent activity in DSB repair.

### Upf1 and SMG6 degrade dilncRNAs

DSB-induced dilncRNAs are bidirectionally synthesized, and their turnover is in part regulated by EXOSC10 (50,51). The mRNA surveillance machinery has a role in degrading nonsense mRNAs in the cytoplasm (28). We examined whether it also regulates the abundance of dilncRNAs at DNA damage sites. We performed RT–qPCR using primers complementary to a site ∼0.5 kb upstream or downstream of the Cas9/sgHPRT-induced cleavage site and observed that depletion of *HOTAIRM1*, Y14, Upf1 or SMG6 increased the level of dilncRNAs to different extents (Figure 7D). DSB-induced transcripts were also detectable at 1 kb but not 40 kb downstream of the cleavage site (Supplementary Figure S7D). Because the primers used were located in the non-coding regions, our observation may lower the possibility of detecting steady-state mRNAs. Furthermore, we used an sgRNA (sgXqCen) targeting the centromere region of the X chromosome q arm (Xq), which is transcriptionally less active (Supplementary Figure S7E). Depletion of *HOTAIRM1* or Y14 reduced the NHEJ activity, suggesting that the *HOTAIRM1* complex also functions in this centromere region (Supplementary Figure S7F). A minimal level of the centromere transcripts was detected upon Cas9-mediated cleavage (Supplementary Figure S7G, lanes 2 and 3). Depletion of Upf1 or SMG6 increased the level of such transcripts (Supplementary Figure S7G), further supporting the role of NMD factors in the degradation of DNA damage-induced transcripts.

Because SMG6 has endonucleolytic activity, we further analyzed whether this activity is essential for DNA repair. Overexpression of siRNA-resistant SMG6 almost fully restored DNA repair in SMG6-depleted cells (Figure 7E, WT-res). The PilT N-terminal (PIN) domain in the C-terminal region of SMG6 is important for its catalytic activity in NMD (56). Overexpression of an SMG6-PIN mutant (D1251A) (36,37) only partially restored DNA repair (Figure 7E, mtPIN-res). Similarly, wild-type, but not mutant, SMG6 partially restored the NHEJ activity in HeLa NHEJ reporter cells (Supplementary Figure S7H). Finally, we examined whether SMG6 is responsible for dilncRNA degradation. Overexpression of wild-type SMG6 reduced the level of DSB-induced dilncRNAs by 15–25%, whereas the mutant increased dilncRNA levels (Figure 7F). Therefore, SMG6 may participate in DSB repair at least in part by degrading dilncRNAs at DSBs.

Together, these results revealed that the mRNA surveillance factors recruited to DSBs by *HOTAIRM1* participate in dilncRNA turnover, which influences the efficiency of DSB repair.

## DISCUSSION

### The role of *HOTAIRM1* in DSB repair

DSB repair involves different types of RNA, including dilncRNA and their processed small RNAs and pre-existing lncRNAs (12,13). In this study, we identified a new lncRNA player, *HOTAIRM1*, in DSB repair. Although our results revealed *HOTAIRM1* as a platform for mRNA surveillance factors and NHEJ factors, the architecture of this ribonucleoprotein complex is as yet unclear. We also identified several *SNHG* lncRNAs in the Y14 immunoprecipitates (Figure 1). In light of two recent reports showing that *SNHG* lncRNAs can participate in DSB repair (26,57), we cannot completely exclude the possibility that Y14-associated *SNHG*s have a role in DNA repair. We provide several lines of evidence for the role of *HOTAIRM1* in DSB repair. First, *HOTAIRM1* interacted with Y14 and NHEJ factors (Figure 2). Laser microirradiation caused *HOTAIRM1* to localize to DNA damage sites (Figure 3). *HOTAIRM1* was essential for efficient recruitment of NHEJ factors and also regulated Ku70 foci dynamics (Figure 4). Finally, *HOTAIRM1* recruited Upf1/SMG6 to DSB sites to regulate dilncRNA turnover (Figure 7).

The association of *HOTAIRM1* with the NHEJ factors is reminiscent of several lncRNAs that have been implicated in NHEJ (26,27,57,58). *LINP1* was the first lncRNA identified, and it acts as a modular scaffold linking Ku and DNA-PK (27). Another study revealed that *LINP1* self-assembles into phase-separated condensates that sequester Ku, and this stabilizes the initial interaction between DNA ends during synapsis and repair (59). Similar to *LINP1*, *NIHCOLE* forms clusters with Ku and promotes ligation of DSBs via recruitment of several NHEJ factors (58). Therefore, potential *NIHCOLE* phase separation may favor repair kinetics. Truncation of the 5′, middle or 3′ part of *HOTAIRM1* impaired its localization to DSB sites to different extents (Figure 3), but its molecular interactions with Y14 and the NHEJ complex still require further investigation. Finally, STRING analysis of the *HOTAIRM1* interactome revealed a number of factors involved in mRNA biogenesis. The question of whether spliceosomal factors regulate R-loops (59) or dilncRNA processing remains to be tested. In the *HOTAIRM1* interactome, RNA polymerase II and the PRP19–CDC5L complex appeared to be a hub (Figure 6). PRP19 not only functions in precursor mRNA splicing but also plays a critical role in multiple DDR signaling networks (60,61). The lncRNA *NORAD* complex also contains PRP19 and contributes to genomic stability (17). Therefore, whether certain lncRNAs, such as *NORAD* and *HOTAIRM1*, share some common factors to coordinate RNA processing and DNA repair warrants further investigation.

Besides pre-existing lncRNAs, DSB-induced dilncRNAs also participate in DNA repair by forming RNA–RNA or DNA–RNA hybrids at DSB sites. dilncRNAs form DNA–RNA hybrids with the resected DNA ends during the S/G_2_ phase of the cell cycle. After degradation of RNA by RNase H2, Rad51 is loaded onto the resultant single-stranded DNA for HR-based repair (24). Using S9.6 antibody immunoprecipitation, we detected a significantly increased level of DNA–RNA hybrids (R-loops) in *HPRT* upon Cas9/sgRNA-mediated cleavage (Supplementary Figure S8A). However, depletion of *HOTAIRM1* had no significant or a minimal effect on R-loop formation (Supplementary Figure S8B), in line with the above observation that the *HOTAIRM1* complex does not contribute to HR (Supplementary Figure S5). Intriguingly, *HOTAIRM1* depletion resulted in Rad51 accumulation on chromatin, suggesting that *HOTAIRM1* may suppress DNA resection (Supplementary Figure S8C). Nevertheless, whether *HOTAIRM1* plays any role in DNA repair pathways rather than NHEJ still needs future studies. dilncRNAs can also be processed into small RNAs in repetitive regions or ribosomal DNA loci via DICER-dependent or -independent pathways (19,22). RNA hybrids formed by dilncRNAs, and those small RNAs can promote the formation of 53BP1-containing DDR foci (19). However, *HOTAIRM1* depletion had no apparent effect on 53BP1 focus formation (Supplementary Figure S4B). Recent evidence indicates that phase-separated condensates of DDR factors provide transient repair compartments for efficient DNA repair. RNA itself has the potential to drive phase separation of DDR factors. For example, inhibition of RNA polymerase II-mediated transcription or disruption of RNA condensation prevents the formation of 53BP1 foci, suggesting a role for dilncRNAs in promoting molecular crowding of DNA repair factors (20). Therefore, whether *HOTAIRM1* self-assembles into phase-separated condensates, as does *LINP* (27), also warrants further investigation.

### The role of mRNA surveillance factors in DSB repair

Upf1 and SMG6 primarily function in NMD in the cytoplasm. Upon association with a stalled ribosome on nonsense mRNAs, Upf1 is phosphorylated by the phosphatidylinositol 3-kinase-related kinase (PIKK) SMG1 and subsequently recruits SMG6 and SMG5/7 to degrade RNA (36,62). SMG1 was not detected in the *HOTAIRM1* ribonucleoprotein complex (Figure 6). Notably, ATM inhibition disrupted the *HOTAIRM1* complex and abolished its localization at DNA damage sites (Figure 3; Supplementary Figure S1). Therefore, it would be interesting to test whether ATM or ATR that also belongs to the PIKK family phosphorylates Upf1 at DNA damage sites. Several early studies have revealed the potential role of Upf1 and SMG6 in maintaining genome/telomere integrity. Upf1 associates with the chromatin during S phase and after DNA damage, whereas SMG6 is a telomerase cofactor (52–54). Notably, a recent report indicated that Upf1 can promote DNA resection and repair at subtelomeric DSBs by driving R-loop formation (55). Our result showing that both Upf1 and SMG6 located to laser-induced DNA damage tracks and that SMG6 shifted from the cytoplasm into the nucleus upon IR treatment emphasizes their general role in DNA damage repair (Figure 7).

Several ribonucleases have been implicated in DNA damage repair. An early study indicated that the yeast exonuclease XRN1 promotes end resection probably by degrading RNAs in the vicinity of DSBs (63). XRN2 plays a role in R-loop resolution and genomic stability (64). More recent studies indicated that DICER and RNase H2, respectively, participate in dilncRNA processing into small RNAs or dilncRNA degradation during S/G_2_ phase (19,24). EXOSC10 degrades dilncRNAs released from DNA–RNA hybrids by the helicase senataxin, leading to replication protein A loading onto resected DNA ends (50,65). In our assay system, depletion of EXOSC10 indeed reduced HR but had no significant effect on NHEJ (Supplementary Figures S5 and S7). Therefore, the function of Upf1/SMG6 in dilncRNA turnover during NHEJ may be analogous to that of senataxin/EXOSC10 during HR. Since depletion of the *HOTAIRM1* complex increased the level of dilncRNAs (Figure 7), we postulate that such dilncRNA accumulation hampers Ku dissociation from DSBs (Figure 4), perhaps similarly to the scenario in which prevention of Ku70/80 ubiquitination or phosphorylation abolishes their dissociation from DSBs and DSB repair (66,67). Therefore, dilncRNA clearance is likely to be a necessary step for DSB repair. It is notable that the RNA moiety of RNA–DNA hybrids at DSBs can be m^6^A-modified by METTL3 upon ATM activation, which facilitates the recruitment of HR factors (68). The *HOTAIRM1* interactome contained several RNA-modifying enzymes (Supplementary Table S6), but whether these enzymes are bona fide components and participate in DSB repair remains to be investigated.

**Figure 8.**
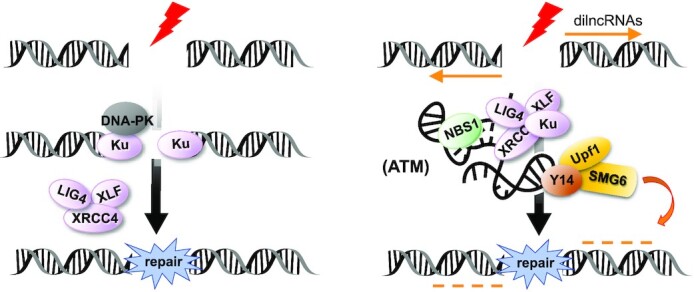
*HOTAIRM1* participates in DSB repair via its assocition with DNA repair and NMD factors. Left: a DSB repair model is depicted without *HOTAIRM1*. Upon DSB induction, the Ku heterodimer forms a complex with DNA-PK, binding to the DNA ends. DNA ligase IV and its cofactor XRCC4 and regulator XLF participate in DNA ligation. This model does not exclude the involvement of RNA. Right: a model shows *HOTAIRM1*-mediated DSB repair. *HOTAIRM1* accumulates at DSBs and is essential for efficient recruitment of NHEJ and NMD factors (Upf1/SMG6) to DSBs and subsequent DSB repair. SMG6 regulates dilncRNA turnover. Depletion of *HOTAIRM1* causes dilncRNA accumulation and may inhibit Ku dissociation from DSB sites. The ATM activity is essential for the integrity of the *HOTAIRM1* ribonucleoprotein complex and its localization at DSB sites.

In conclusion, we uncovered the *HOTAIRM1* ribonucleoprotein complex and demonstrated its role in NHEJ-mediated DSB repair. ATM signaling is required for its integrity and localization at DNA damage sites, where NMD factors participate in dilncRNA turnover (Figure 8). Nonetheless, many questions, such as whether different ribonucleases function in different repair pathways and whether Upf1/SMG6 particularly degrade aborted transcripts upon DNA damage, still remain to be investigated.

## DATA AVAILABILITY

The RNA-seq data generated in this study have been deposited in the NCBI SRA database with the BioProject accession ID PRJNA827119, https://www.ncbi.nlm.nih.gov/bioproject/827119. The mass data generated in this study have been deposited in the ProteomeXchange Consortium via the PRIDE (https://www.ebi.ac.uk/pride) partner repository with the dataset identifier PXD034470].

## Supplementary Material

gkad143_Supplemental_Files
