# Supplementary material for: LncRNA HOTAIRM1 functions in DNA double-strand break repair via its association with DNA repair and mRNA surveillance factors
Source: Nucleic Acids Res. 2023 Mar 7;51(7):3166–84. doi: 10.1093/nar/gkad143 (PMC10123106; doi:10.1093/nar/gkad143)
Supplement: gkad143_Supplemental_Files [file gkad143_supplemental_files.zip › Supplmental_Information.pdf]

SUPPLEMENTAL INFORMATION

lncRNA *HOTAIRM1* Functions in DNA Double-Strand Break Repair via its Association with DNA Repair and mRNA Surveillance Factors

Tzu-Wei Chuang[1], Chun-Hao Su[1], Pei-Yu Wu[2], Yao-Ming Chang[1] and Woan-Yuh Tarn[1,*]
[1]Institute of Biomedical Sciences, Academia Sinica, Taipei, Taiwan
[2]Institute of Biological Chemistry, Academia Sinica, Taipei, Taiwan

*Corresponding author:
Woan-Yuh Tarn, Ph.D.
Institute of Biomedical Sciences
Academia Sinica
128 Academy Road Section 2
Nankang, Taipei 11529
Taiwan
Telephone: 8862-2652 3052
FAX: 8862-2782 9142
E-mail: wtarn@ibms.sinica.edu.tw

SUPPLEMENTAL MATERIALS AND METHODS

Cell Culture

For DNA damage induction, HeLa, HEK293 or U2OS cells were irradiated with X-rays (10 Gy) or treated with phleomycin (500 μM, InvivoGen) or Zeocin (500 μg/ml, InvivoGen) for 2 hours. Cells were treated with DNA repair inhibitors as follows: ATM inhibitor KU-55933 (10 μM, Tocris) for 1 hour, DNA-PK inhibitor NU7441 (10 μM, Tocris) for 1 hour or MRE11 inhibitor Mirin (500 μM, Sigma) for 2 hours.

Plasmids

The pEGFP-C1-FLAG-Ku80 was purchased from Addgene. Ku80ΔCD lacking the central domain (amino acid 268-539) was generated by using a PCR-based method. The pcDNA-β-globin-6×MS2 vector was provided by Jens Lykke-Andersen. For *in vitro* transcription, the cDNA encoding full-length *HOTAIRM1* or fragments was inserted into the pGEM-T vector (Promega). The flanking sequence was shortened by restriction digestion with NsiI/NotI, following by blunt-ending and re-ligation. The CRISPR/Cas9-sgRNA fusion vector (sgXqCen) is a kindly gift from Patrick Ching-Ho Hsieh. In briefly, the sgRNA was targeting to upstream sequence of *spindling family member 4 (SPIN4)* on human X chromosome, which is nearby the centromere. The sequence of all the constructs was confirmed by Sanger sequencing.

Electrophoretic Mobility Shift Assay (EMSA)

For *in vitro* transcription, the pGEM-HM1 (*HOTAIRM1*, 632-882) vector was linearized by SacII. In vitro transcription was performed using by Sp6 RNA polymerase (Promega) according to the manufacturer's instructions. The reaction contained 0.5 mM ATP, CTP and GTP, 0.1 mM UTP and 3.3 μM of α-^{32}P-UTP (3000 Ci/mmol, 10 mCi/ml, PerkinElmer). RNA was gel purified. The specific radioactivity of was 3×10^6 cpm/μg. For EMSA, 5×10^4 cpm ^{32}P-labeled HM1.4 was incubated with Y14 in a buffer containing 50 mM HEPES (pH 7.0), 150 mM NaCl, 1 mM DTT, 10% glycerol, 0.5 mg/ml BSA on ice for 20 min. The reaction was mixed with the loading dye containing 0.01% xylene cyanol, 0.01% bromophenol blue and separated by electrophoresis on a 4% polyacrylamide gel in Tris-Borate-EDTA buffer. Gels were subjected to fluorography using X-ray film.

Proximity Ligation Assay

For the *in situ* proximity ligation assay, U2OS cells were treated with phleomycin (500 μM), fixed with 4% paraformaldehyde, permeabilized in 0.5 % Triton X-100 and then blocked in Duolink Blocking Solution (Sigma) at 37°C for 1 hour. After hybridization with biotinylated NSO, *HOTAIRM1* or *Gas5* oligonucleotide (Table S8) at 37°C for 24 hrs, cells were incubated with anti-γH2AX and anti-Biotin in Duolink Antibody Diluent (Sigma) at 4°C for 24 hours. Following incubation with PLUS and MINUS PLA probes (Sigma) in Duolink Antibody Diluent at 37 °C for 1 hr, cells were incubated with the Ligation Solution at 37 °C for 1 hr. Ligation was followed by incubation with Amplification Solution at 37 °C for 100 minutes. After final washes, cells were mounted with Duolink In Situ PLA Mounting Media with DAPI (Sigma) and analyzed in a confocal microscope (LSM 780, Carl Zeiss).

Immunofluorescence

For immunofluorescence of 53BP1, U2OS cells were transiently transfected with GapmeR (gNSO or gHM1) for 48 hours. After 10 Gy IR treatment, cells were fixed with 4% paraformaldehyde and permeabilized in 0.5 % Triton X-100 in PBS. Then cells were incubated with antibodies against 53BP1 followed by incubation with Alexa Fluor 568-conjugated anti-rabbit IgG (Thermo Fisher Scientific). Nuclei were counterstained in Mounting Medium with DAPI. Samples were visualized using a laser-scanning confocal microscope (Zeiss LSM880 Airyscan Confocal microscope, Carl Zeiss) coupled with an image analysis system.

Cell Cycle Analysis with Flow Cytometry

HeLa cells were transfected with gNSO or gHM1. Forty eight hrs post-transfection, cells were collected and washed with cold PBS and fixed in 70% ethanol overnight at -20°C. Following, cells were pelleted by centrifugation, resuspended in PBS for 15 min and then stained in PBS containing 20 μg/ml propidium iodide, 0.1% triton X-100 and 0.2 mg/ml RNase A. The samples were kept in the dark at room temperature for 15 min. Cell cycle analysis was performed on an LSRII SORP-17 color analyzer (Becton Dickinson, BD); the data were analyzed by using FlowJo v10 software (BD). For DNA damage sensitivity analysis, cells were transfected as above and then irradiated with X-ray at 5 Gy. The sub-G1 population was analyzed by using flow cytometry.

HR Assay

To assay the HR activity, a single stranded DNA oligonucleotide ssODN (20 pmole, GENOMICS) (1) was co-transfected with the Cas9/sgHPRT expressing vector and siRNAs or GapmeRs into HeLa cells. Forty-eight hrs post-transfection, genomic DNA was collected for qPCR using specific primers and PerfeCTa SYBR Green FastMix PCR Reagent (Quanta Biosciences) in a LightCycler 480 Real-Time PCR System (Roche).

Sucrose Gradient Sedimentation

HEK293 cells (~1×10^7) were collected in PBS containing 100 μg/ml cycloheximide and centrifuged. The cell pellet was resuspended and lysed in a buffer containing 10 mM Tris-HCl, pH 7.4, 3 mM $MgCl_2$, 150 mM NaCl, 100 μg/ml cycloheximide, 40 μg/ml digitonin, 20 U/ml RNasin (Invitrogen) and protease inhibitor (Roche) for 5 min at 4°C. The cell lysates were centrifuged at 3,000×*g* for 1 min and the supernatant were further centrifuged at 11,000×*g* for 15 min. The supernatant (~450 μl) was loaded onto a linear gradient of 15 to 40% sucrose and centrifuged at 36,000 rpm in a Beckman SW41 rotor at 4°C for 3 h. The gradient was collected with 0.5 ml per fraction from top to bottom. RNA was extracted by using Trizol LS (Invitrogen) following the manufacturer's instructions. 18S and 28S rRNA and *HOTAIRM1* were detected by RT-PCR.

DNA-RNA Hybrid Immunoprecipitation (DRIP) Assay

DRIP was adapted from Chuang et al. (2). HeLa cells were mock-transfected or transfected with the Cas9/sgHPRT vector alone with GapmeR (gNSO or gHM1) for 48 hours. The transfected cells were harvested by lysis buffer (1X TE, 0.05% SDS and 0.05 mg/mL Proteinase K) and incubated at 37°C overnight. Total genomic DNA was extracted by standard phenol-chloroform extraction procedure and fragmented by bioruptor sonication device to

approximately 300 bp. Samples were mock-treated or treated with 10 U RNase H (New England Biolabs) at 37°C overnight before DRIP. Subsequently, 5 μg of genomic DNA was incubated with 2.5 μg of S9.6 antibody (Millipore) in the immunoprecipitation buffer (1x PBS pH 7.4 containing 0.05% Triton-X-100) at 4°C for 4 hours with rotation, followed by immunoprecipitation using Protein A Sepharose™ CL-4B (Cytiva) at 4°C for 2 hours. Beads were washed with the immunoprecipitation buffer three times for 10 min each. Bound fractions were eluted with elution buffer (50 mM Tris-HCl pH 8.0, 10 mM EDTA pH 8.0 and 0.5% SDS) at 37°C for 10 min followed by phenol-chloroform extraction. Finally, qPCR was performed using primer targeting upstream of *HPRT* (Table S8).

Copy Number of *HOTAIRM1*

The copy number of *HOTAIRM1* in U2OS cells was estimated by RT-qPCR using *in vitro* transcribed *HOTAIRM1* RNA as standard.

Nuclear and Cytosolic Fractionation

U2OS cells were treated with or without IR and then washed with PBS, followed by resuspending in the hypotonic buffer (20 mM Tris-HCl pH 7.4, 10 mM KCl, 2 mM $MgCl_2$, 1 mM EGTA, 0.5 mM DTT and 0.5 mM PMSF). After incubation on ice for 3 min, 0.1% NP-40 was added for additional 3 min incubation. Cell lysate was centrifuged at 1,000×*g* for 5 min at 4°C. The cytosolic fraction was collected after centrifugation at 15,000 ×*g* for 3 min at 4°C. To collect the nuclear fraction, the cell pellet was resuspended in the isotonic buffer containing 20 mM Tris-HCl pH 7.4, 150 mM KCl, 2 mM $MgCl_2$, 1 mM EGTA, 0.5 mM DTT and 0.5 mM PMSF with 0.1% NP-40 on ice for 5 min. After centrifugation at 1,000 ×*g* for 3 min at 4°C, the supernatant was collected.

SUPPLEMENTAL FIGURE LEGENDS

Figure S1. Characterization of the Interaction Between Y14/NHEJ Factors and *HOTAIRM1*. Related to Figure 2.

(A) HEK293 cells were transiently transfected with the vector expressing the indicated Y14 version. Cell fractionation was performed as previously described (2). Immunoblotting was performed using antibodies against indicated proteins. S, soluble fraction (cytosolic and nucleoplasmic fractions); C, chromatin fraction. Numbers below the gels indicate relative abundance of each Y14 version in the chromatin-enriched fraction.

(B) Diagram shows Ku80 and central domain-truncated Ku80 (Ku80ΔCD). HEK293 cells were transiently transfected with the empty vector (vec) or vector expressing GFP-FLAG-Ku80 (full-length or ΔCD), followed by immunoprecipitation and RT-PCR (*HOTAIRM1* and *ACTB*) or immunoblotting using anti-FLAG and anti-Ku70.

(C) Different concentrations of recombinant Y14 or Ku70/80 as in Figure 2D was incubated with ^{32}P-labeled *HOTAIRM1* 632-882, and RNA-protein complexes were fractionated in non-denaturing gels.

(D) Secondary structure of *HOTAIRM1* was predicted by RNAfold Webserver (rna.tbi.univie.ac.at). All types of antisense oligonucleotides used in this study are indicated as in Figure 2E.

(E) Immunoprecipitation of transiently expressed FLAG-Y14 was performed as in Figure 2F. ASOs were each added into anti-FLAG immunoprecipitates but without RNase H treatment.

(F) Affinity selection was performed as in Figure 2G using three different bHM1 oligonucleotides. Immunoblotting and RT-PCR was performed as in Figure 2G.

(G) Affinity selection of *HOTAIRM1* and *Gas5* was performed using biotinylated oligonucleotides as indicated, followed by immunoblotting and RT-PCR.

(H) Affinity selection was performed as in Figure 2G. bHM1-selected proteins were mock-treated (lane 4) or treated with DNase (lane 5), followed by immunoblotting. *HOTAIRM1* and *ACTB* were detected by RT-PCR.

(I) HeLa cells were transfected with empty vector (vec) or GFP-FLAG-Ku70 vector or together with the indicated GapmeR. Anti-FLAG immunoprecipitates were subjected to immunoblotting and RT-PCR for indicated protein or RNA.

(J) Affnity selection of *HOTAIRM1* was performed as in Figure 2G from the HEK293 cell lysate that was respectively treated with IR and/or ATM inhibitor KU-55933 (ATMi). Selected proteins were subbjected to immunoblotting.

Figure S2. The Effect of *HOTAIRM1* Depletion in Cell Cycle. Related to Figure 2.

(A) HeLa cells were transfected with gNSO or gHM1. Cells were stained with propidium iodide (PI) and the DNA content was determined by flow cytometry. A representative result is shown at left. Bar graphs show percentage of cells at each cell cycle phase.

(B) The experiment was performed as in panel A. Bar graph shows percentage of cells in sub-G1 population.

(C) Bar graph shows percentage of sub-G1 population in gNSO or gHM1-transfected cells that were mock treated or irradiated by 5-Gy X-ray. All bar graphs show mean ± SD; N=3.

Figure S3. Localization of *HOTAIRM1* after DNA damage. Related to Figure 3.

(A) HeLa cells were transfected with siC or siKu80, followed by immunoblotting analysis.

(B) U2OS cells were mock-treated or treated with DNA-PK inhibitor (NU7441) or MRN inhibitor (Mirin). FISH images and bar graph show the effect of these inhibitors on *HOTAIRM1* localization at DNA damage sites (mean ± SD; N=3).

(C) RT-qPCR of *HOTAIRM1* was performed in the cytosol (C) and nuclear (N) fractions of U2OS cells without or after IR. Bar graph shows the relative level of nuclear to cytoplasmic *HOTAIRM1* (cytosol was set to 1; mean ± SD; N=3). IR increased the relative level of *HOTAIRM1* in the nucleus. Immunoblotting indicates subcellular fractionation using lamin A/C and GAPDH as markers.

(D) HeLa cells were mock-treated or treated with IR, phleomycin or zeocin. Soluble fraction (S, including cytosolic and nucleoplasmic fractions) and chromatin fraction (C) were subjected to RT-PCR (*HOTAIRM1*) and immunoblotting (GAPDH and histone 3). Numbers below the gel indicate the increase fold of *HOTAIRM1* in the chromatin-enriched fraction after treatment. (N=3)

(E) Total cellular RNAs resepctively from 5×10^4 and 10^5 U2OS cells were subjected to RT-qPCR using *in vitro* transcribed *HOTAIRM1* (0.2, 2 and 20 pg) as standard. Bar graph shows the number of *HOTAIRM1* molecules (the y-axis).

(F) Diagram shows MS2-tagged full-length and truncated *HOTAIRM1* and primers used for RT-qPCR. Bar graph shows the relative level of overexpressed MS2-*HOTAIRM1* to endogenous *HOTAIRM1*. For truncated versions, RT-qPCR was performed using the primer combinations as indicated (colored: endogenous; black: overexpressed). For the

full-length *HOTAIRM1*, the level of the endogenous was estimated by subtraction of the overexpressed (black) from the total (blue) (mean ± SD; N=3).

(G) As shown in Figure 3D, microirradiation was performed in U2OS cells that expressed MS2 or β-globin-MS2. Localization of RNA was detected by GFP-MCP. γH2AX signals indicated DNA damage tracks. RT-PCR revealed the expression of MS2 alone and MS2-tagged *HOTAIRM1* or β-globin. Scale bar, 10 μm.

(H) U2OS cells were treated with phleomycin followed by proximity-ligation assay. RNA was hybridized by using biotinylated NSO, *HOTAIRM1* or *Gas5* oligonucleotide. Dots represent RNA-γH2AX interaction. Bar graph shows proximity-ligation assay dots quantified from 18-20 cells per sample. Scale bar, 10 μm.

(I) RT-PCR was performed in cell lines as indicated using primers complementary to exons 1 and 3.

(J) HEK293 cells were transiently transfected with vector (mock or expressing FLAG-Y14- or GFP-FLAG-Ku70) as in Figure 2C, followed by RT-PCR (two isoforms of *HOTAIRM1, DANCR and ACTB*).

Figure S4. Depletion of *HOTAIRM1* has no Effect on γH2AX Foci Formation. Related to Figure 4.

(A) U2OS cells were transfected and irradiated as in Figure 4B. Bar graph shows the relative intensity of γH2AX foci; gNSO (-IR) was set to 1; for each sample, 9-11 cells were measured (mean ± SD).

(B) Immunofluorescence of 53BP1 was performed in mock-depleted or *HOTAIRM1*-depleted U2OS cells without and after IR. Bar graph shows that relative intensity of γH2AX and 53BP1 foci; for each sample, 16-18 cells were measured (mean ± SD). RT-PCR showed the efficiency of *HOTAIRM1* depletion. Scale bar, 20 μm.

Figure S5. Depletion of the *HOTAIRM1* components has no effect on HR. Related to Figure 5.

(A) Diagram shows Cas9/sgHPRT-mediated cleavage and HR repair assay (1). A ssDNA oligonucleotide was used as the template for HR, containing the Ins sequence (green as in Figure 5A) in the center and the sequences complementary to the cleavage region at the 5' and 3' ends (grey).

(B) The Cas9/sgHPRT vector, ssDNA template, and siRNA/GapmeR as indicated were co-transfected in HeLa cells. Mirin represents MRN inhibitor, which can inhibit HR (1). qPCR was performed using the primer sets I-R and F-R; the F-R PCR product was used for normalization. Bar graph shows the relative HR repair efficiency (sg+Ins was set to 1; mean ± SD; N=3). Immunoblotting and RT-PCR respectively show depletion efficiency of Y14 and EXOSC10, and *HOTAIRM1.*

Figure S6. Characterization of *HOTAIRM1* and its Interaction with DNA Repair and RNA Processing Factors. Related to Figure 6.

(A) HEK293 cell lysate was subjected to 15-40% sucrose gradient sedimentation. RNA was recovered from the 24 fractions for RT-PCR analysis (rRNA and *HOTAIRM1*). Dots denote potential association with *HOTAIRM1* with the 40S ribosomal subunit or the 80S ribosome.

(B) Affinity selection of *HOTAIRM1* and *Gas5* was performed using biotinylated oligonucleotides as in Figures 2G and S1F, followed by immunoblotting and RT-PCR.

(C) HEK293 cells were mock-irradiated (–IR) or irradiated (10 Gy; +IR). Cell lysates were incubated with bNSO and bHM1, followed by affinity selection using streptavidin. Immunoblotting and RT-PCR were performed using specific antibodies or primers as indicated.

(D) HEK293 cells were transfected with the empty or FLAG-Y14 vector. FLAG-Y14-containing cell lysates were mock-treated or treated with RNase A. Cell lysates (vec or FLAG-Y14 without or with RNase A treatment) were subjected to anti-FLAG-immunoprecipitation and immunoblotting.

Figure S7. Upf1 and SMG6 are Essential for Repair of I-SceI-cleaved DSB. Related to Figure 7.

(A) HeLa cells were treated with IR. Chromatin fraction collected at indicated time points after IR was subjected to immunoblotting.

(B) DSB repair assay was performed as in Figure 5D. HeLa NHEJ reporter cells were transfected with pSCE and siRNA as indicated. Percentage of GFP-positive cells was measured by flow cytometry (mean ± SD; N=5; $^{***}p < 0.001$).

(C) The Cas9/sgHPRT-based NHEJ assay was performed in HeLa cells that were transfected with single or double siRNA or siRNA/GapmeR as indicated. The relative NHEJ activity was measured as in Figure 5B (sg+Ins was set to 1; mean ± SD; N=3). Immunoblotting and RT-PCR respectively show protein and *HOTAIRM1* depletion efficiency.

(D) Diagram shows three sets of primers complementary to the region at approximately 0.5 kb (blue), 1 kb (green) and 40 kb (red) downstream of the Cas9/sgHPRT-mediated cleavage site; all primers were located in the intron or non-coding regions. The experiment was performed as in Figure 7D. Bar graph shows the relative level of dilncRNA transcripts as in Figure 7D (data of 0.5 kb are shown in Figure 7D) (mean ± SD; N=6).

(E) Diagram shows Cas9/sgXqCen targeting a site between the centromere region and the *SPIN4* gene of the X chromosome q arm.

(F) As in Figure 5A, experimental design for Cas9/sgXqCen-mediated cleavage and repair. The Cas9/sgXqCen vector was co-transfected with the Ins dsDNA and siRNA/GapmeR respectively targeting Y14 or *HOTAIRM1.* The level of PCR products (I+R) was normalized to that of F+R; mean values of the relative DNA repair efficiency (siY14/siC and gHM1/gNSO) were indicated below the gel (N=3).

(G) The Cas9/sgXqCen vector was co-transfected with siRNA targeting Y14, Upf1 or SMG6. RT-(q)PCR was performed using the primer set (green arrows) upstream of the cleavage site, *i.e.* in the centromere region. RT-PCR products were shown in the gel. Bar graphs show relative levels of dilncRNAs (mean ± SD; N=3).

(H) As in panel A, HeLa NHEJ reporter cells were transfected with pSCE and siC or siSMG6 and SMG6 expression vector (WT-res or mtPIN-res). GFP measurement and bar graph were as in panel A (mean ± SD; N=3; $^{***}p < 0.001$). Immunoblotting in both panels shows knockdown or overexpression of indicated proteins.

Figure S8. The effect of *HOTAIRM1* depletion on Rad51 and R-loops. Related to Discussion.

(A) Diagram shows Cas9/sgHPRT-targeting site and potential R-loop formation. Primers used for DNA-RNA immunoprecipitation (DRIP) are indicated. HeLa cells were mock-transfected or transfected with the Cas9/sgHPRT vector. Genomic DNA was collected and mock-treated or treated with RNase H followed by immunoprecipitation using IgG or S9.6 antibody; precipitated DNA-RNA hybrids were subjected to qPCR (mean ± SD; N=3).

(B) HeLa cells were transfected with the Cas9/sgHPRT vector and GapmeR (gNSO or gHM1). DRIP and bar graph were as shown in panel A. N=3.

(C) HeLa cells transfected with gNSO or gHM1 were treated with IR. Total lystate and chromatin fractionation were collected at indicated time after IR, followed by immunoblotting.

SUPPLEMENTAL TABLES

Tables S1-S6, see Excel files

Table S1. List of Y14-Associated Transcripts Identified by RIP-seq. Related to Figure 1A.

Table S2. Reactome Pathway Enrichment Analysis of Proteins Encoded by Y14-Associated mRNAs. Related to Figure 1B.

Table S3. List of *HOTAIRM1*-Associated Proteins Identified by Mass Spectrometry. Related to Figure 6A.

Table S4. List of *HOTAIRM1*-Associated Proteins with ≥ 5 Protein Member Hits in the MASCOT Search. Related to Figure 6B.

Table S5. KEGG Pathway Enrichment Analysis of *HOTAIRM1*-Associated Proteins that are Listed in Table S4. Related to Figure 6B.

Table S6. List of *HOTAIRM1*-Associated Proteins that Function in mRNA Biogenesis and DNA Damage Repair. Related to Figure 6C.

Table S7. List of Antibodies Used for Immunoblotting and Immunofluorescence

Table S8. List of siRNAs, Antisense Oligonucleotides, GapmeRs and Biotinylated Oligonucleotides and Primers Used for PCR and RT-PCR

REFERENCES

1. Du, J., Yin, N., Xie, T., Zheng, Y., Xia, N., Shang, J., Chen, F., Zhang, H., Yu, J. and Liu, F. (2018) Quantitative assessment of HR and NHEJ activities via CRISPR/Cas9-induced oligodeoxynucleotide-mediated DSB repair. *DNA Repair (Amst)*, **70**, 67-71.
2. Chuang, T.W., Lu, C.C., Su, C.H., Wu, P.Y., Easwvaran, S., Lee, C.C., Kuo, H.C., Hung, K.Y., Lee, K.M., Tsai, C.Y. *et al.* (2019) The RNA Processing Factor Y14 Participates in DNA Damage Response and Repair. *iScience*, **13**, 402-415.

FigS1

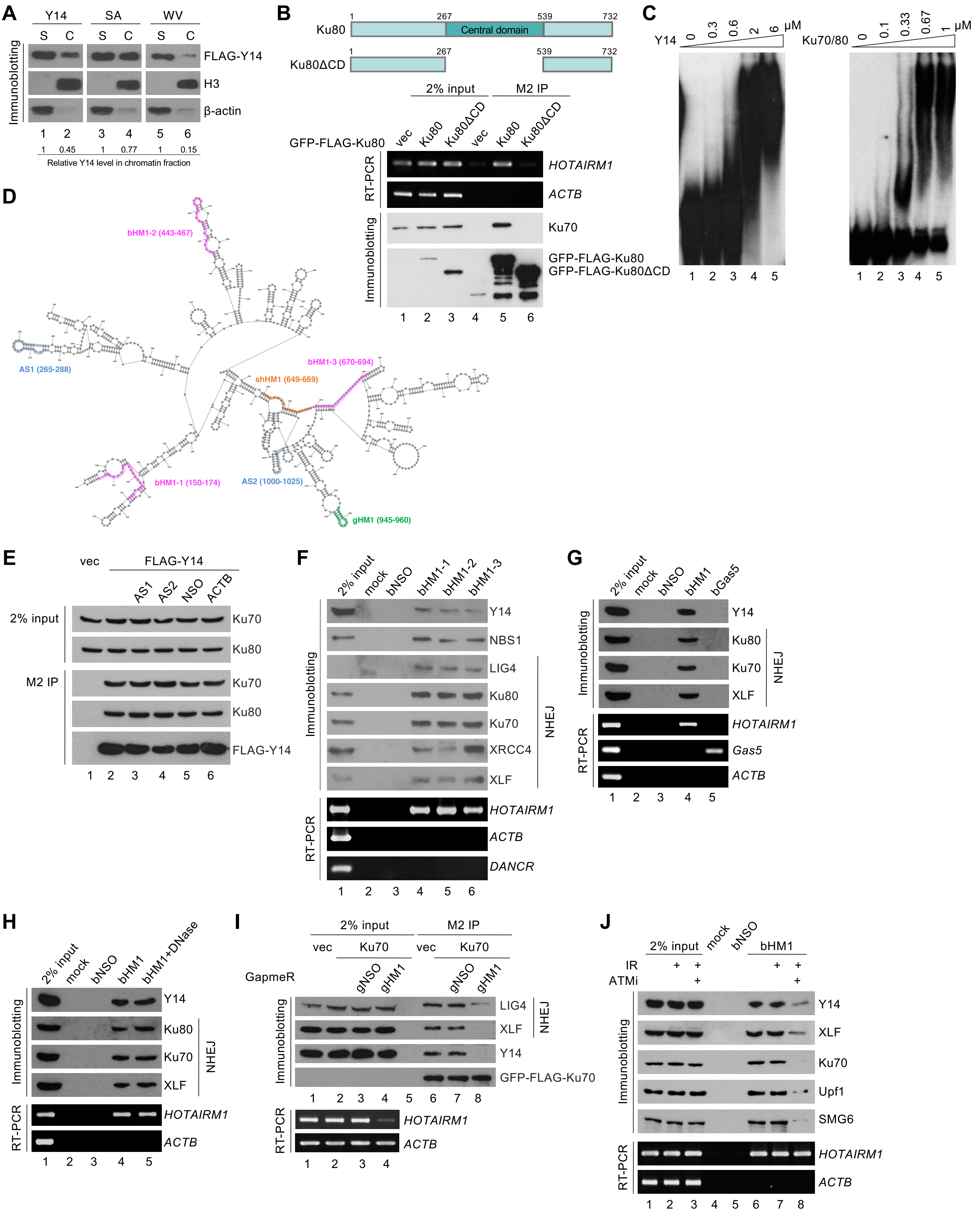
A
Y14
SA
WV
S
C
Immunoblotting
FLAG-Y14
H3
β-actin
1 2 3 4 5 6
1 0.45 1 0.77 1 0.15
Relative Y14 level in chromatin fraction
B
Ku80
Ku80ΔCD
1
267
539
732
Central domain
2% input
M2 IP
GFP-FLAG-Ku80
vec
Ku80
Ku80ΔCD
RT-PCR
HOTAIRM1
ACTB
Immunoblotting
Ku70
GFP-FLAG-Ku80
GFP-FLAG-Ku80ΔCD
1 2 3 4 5 6
C
Y14
0 0.3 0.6 2 6 μM
Ku70/80
0 0.1 0.33 0.67 1 μM
1 2 3 4 5
D
bHM1-2 (443-467)
AS1 (265-288)
bHM1-3 (670-694)
shHM1 (649-669)
bHM1-1 (150-174)
AS2 (1000-1025)
gHM1 (945-960)
E
vec
FLAG-Y14
AS1
AS2
NSO
ACTB
2% input
M2 IP
Ku70
Ku80
FLAG-Y14
1 2 3 4 5 6
F
2% input
mock
bNSO
bHM1-1
bHM1-2
bHM1-3
Immunoblotting
Y14
NBS1
LIG4
Ku80
Ku70
XRCC4
XLF
NHEJ
RT-PCR
HOTAIRM1
ACTB
DANCR
1 2 3 4 5 6
G
2% input
mock
bNSO
bHM1
bGas5
Immunoblotting
Y14
Ku80
Ku70
XLF
NHEJ
RT-PCR
HOTAIRM1
Gas5
ACTB
1 2 3 4 5
H
2% input
mock
bNSO
bHM1
bHM1+DNase
Immunoblotting
Y14
Ku80
Ku70
XLF
NHEJ
RT-PCR
HOTAIRM1
ACTB
1 2 3 4 5
I
2% input
M2 IP
vec
Ku70
GapmeR
gNSO
gHM1
Immunoblotting
LIG4
XLF
NHEJ
Y14
GFP-FLAG-Ku70
1 2 3 4 5 6 7 8
RT-PCR
HOTAIRM1
ACTB
1 2 3 4
J
2% input
mock
bNSO
bHM1
IR
ATMi
+
Immunoblotting
Y14
XLF
Ku70
Upf1
SMG6
RT-PCR
HOTAIRM1
ACTB
1 2 3 4 5 6 7 8

FigS2

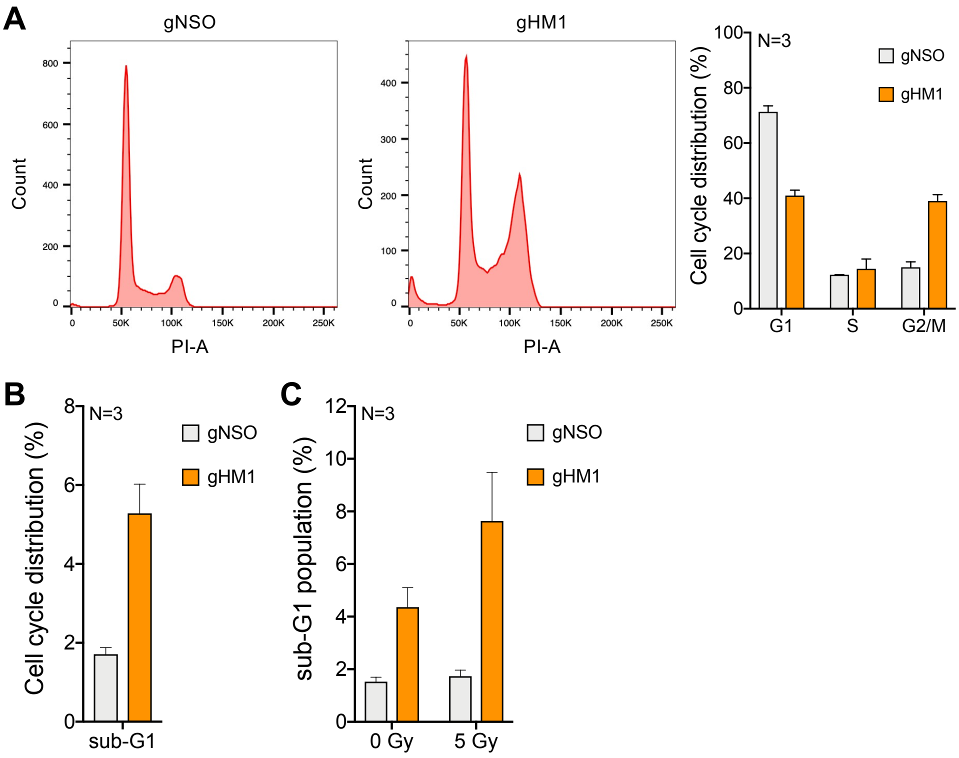
A
gNSO
gHM1
Count
PI-A
0
50K
100K
150K
200K
250K
200
400
600
800
100
300
N=3
gNSO
gHM1
Cell cycle distribution (%)
0
20
40
60
80
100
G1
S
G2/M
B
N=3
gNSO
gHM1
Cell cycle distribution (%)
0
2
4
6
8
sub-G1
C
N=3
gNSO
gHM1
sub-G1 population (%)
0
2
4
6
8
10
12
0 Gy
5 Gy

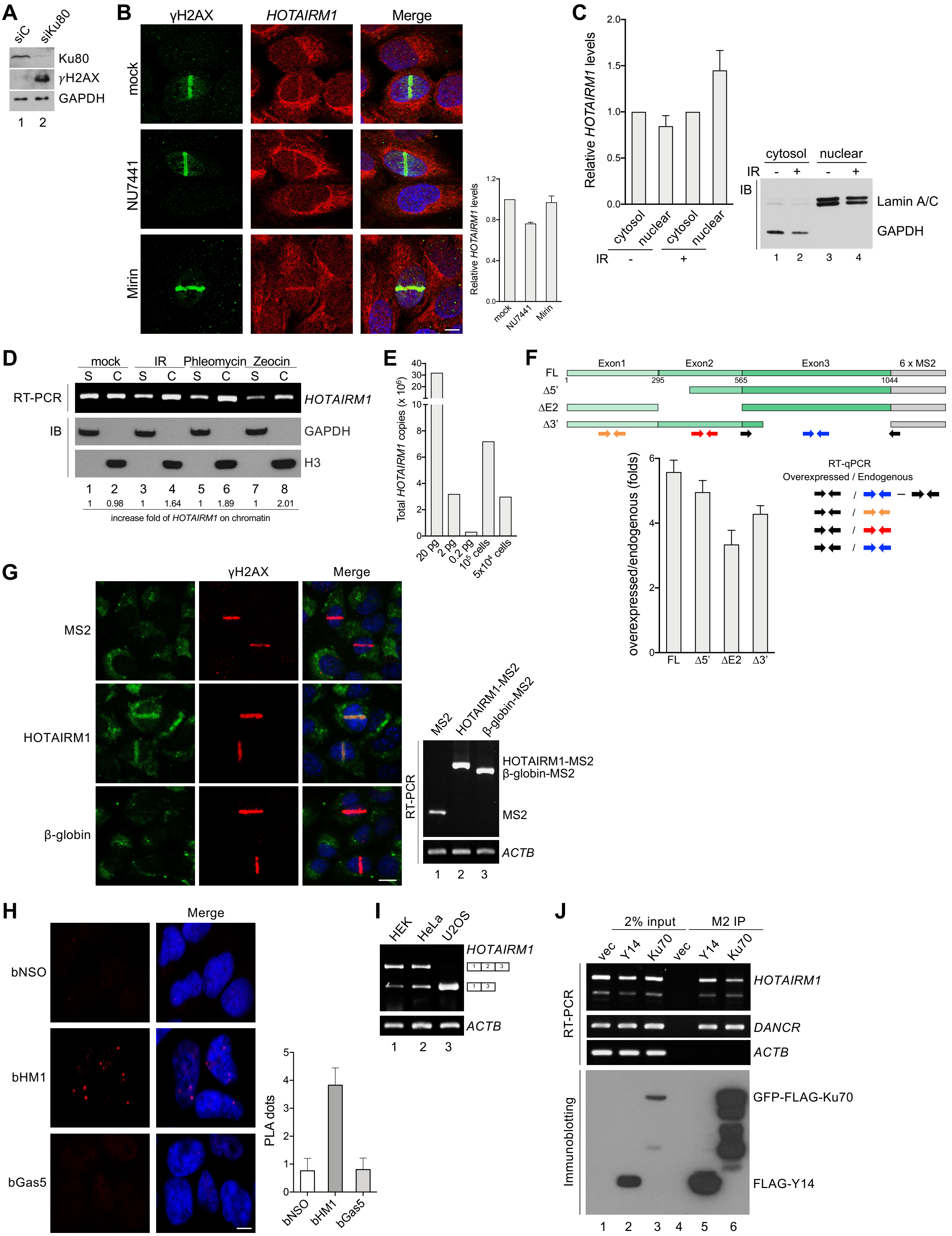
A
siC
siKu80
Ku80
γH2AX
GAPDH
1 2
B
γH2AX
HOTAIRM1
Merge
mock
NU7441
Mirin
Relative HOTAIRM1 levels
C
Relative HOTAIRM1 levels
cytosol
nuclear
IR
IB
Lamin A/C
GAPDH
1 2 3 4
D
mock
IR
Phleomycin
Zeocin
S C S C S C S C
RT-PCR
HOTAIRM1
IB
GAPDH
H3
1 2 3 4 5 6 7 8
1 0.98 1 1.64 1 1.89 1 2.01
increase fold of HOTAIRM1 on chromatin
E
Total HOTAIRM1 copies (x 10^6)
20 pg
2 pg
0.2 pg
10^5 cells
5x10^4 cells
F
Exon1
Exon2
Exon3
6 x MS2
FL
Δ5'
ΔE2
Δ3'
1
295
565
1044
overexpressed/endogenous (folds)
RT-qPCR
Overexpressed / Endogenous
G
γH2AX
Merge
MS2
HOTAIRM1
β-globin
HOTAIRM1-MS2
β-globin-MS2
RT-PCR
ACTB
1 2 3
H
Merge
bNSO
bHM1
bGas5
PLA dots
I
HEK
HeLa
U2OS
HOTAIRM1
ACTB
1 2 3
J
2% input
M2 IP
vec
Y14
Ku70
RT-PCR
HOTAIRM1
DANCR
ACTB
Immunoblotting
GFP-FLAG-Ku70
FLAG-Y14
1 2 3 4 5 6

FigS4

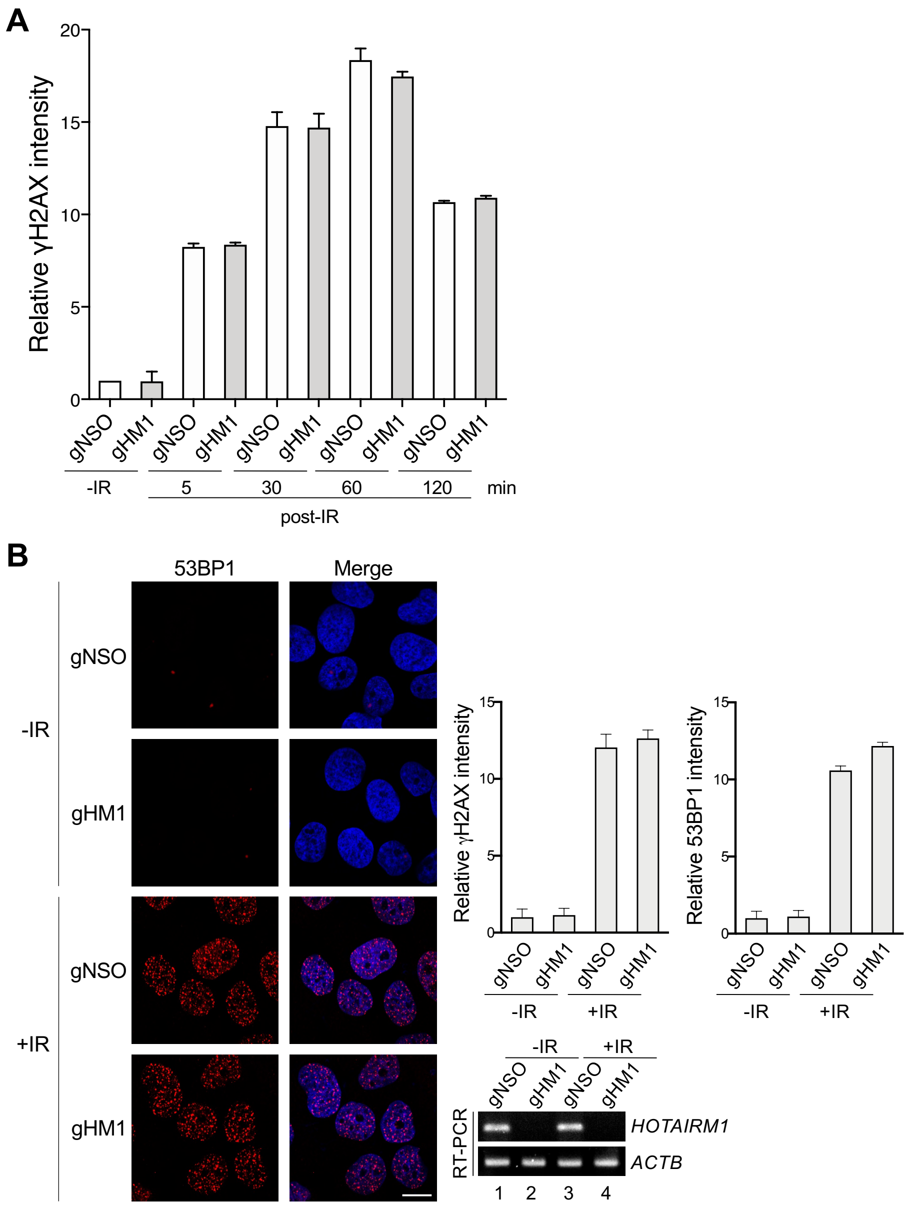
A
Relative γH2AX intensity
20
15
10
5
0
gNSO
gHM1
-IR
5
30
60
120
min
post-IR
B
53BP1
Merge
-IR
+IR
gNSO
gHM1
Relative γH2AX intensity
Relative 53BP1 intensity
RT-PCR
HOTAIRM1
ACTB
1 2 3 4

FigS5

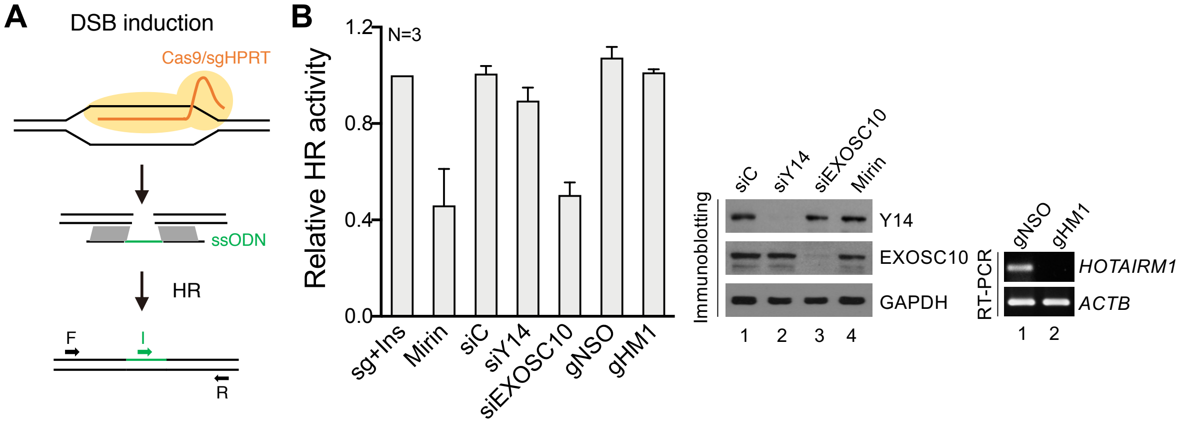

A
DSB induction
Cas9/sgHPRT
ssODN
HR
F
I
R
B
N=3
Relative HR activity
1.2
0.8
0.4
0.0
sg+Ins
Mirin
siC
siY14
siEXOSC10
gNSO
gHM1
Immunoblotting
siC
siY14
siEXOSC10
Mirin
Y14
EXOSC10
GAPDH
1
2
3
4
RT-PCR
gNSO
gHM1
HOTAIRM1
ACTB
1
2

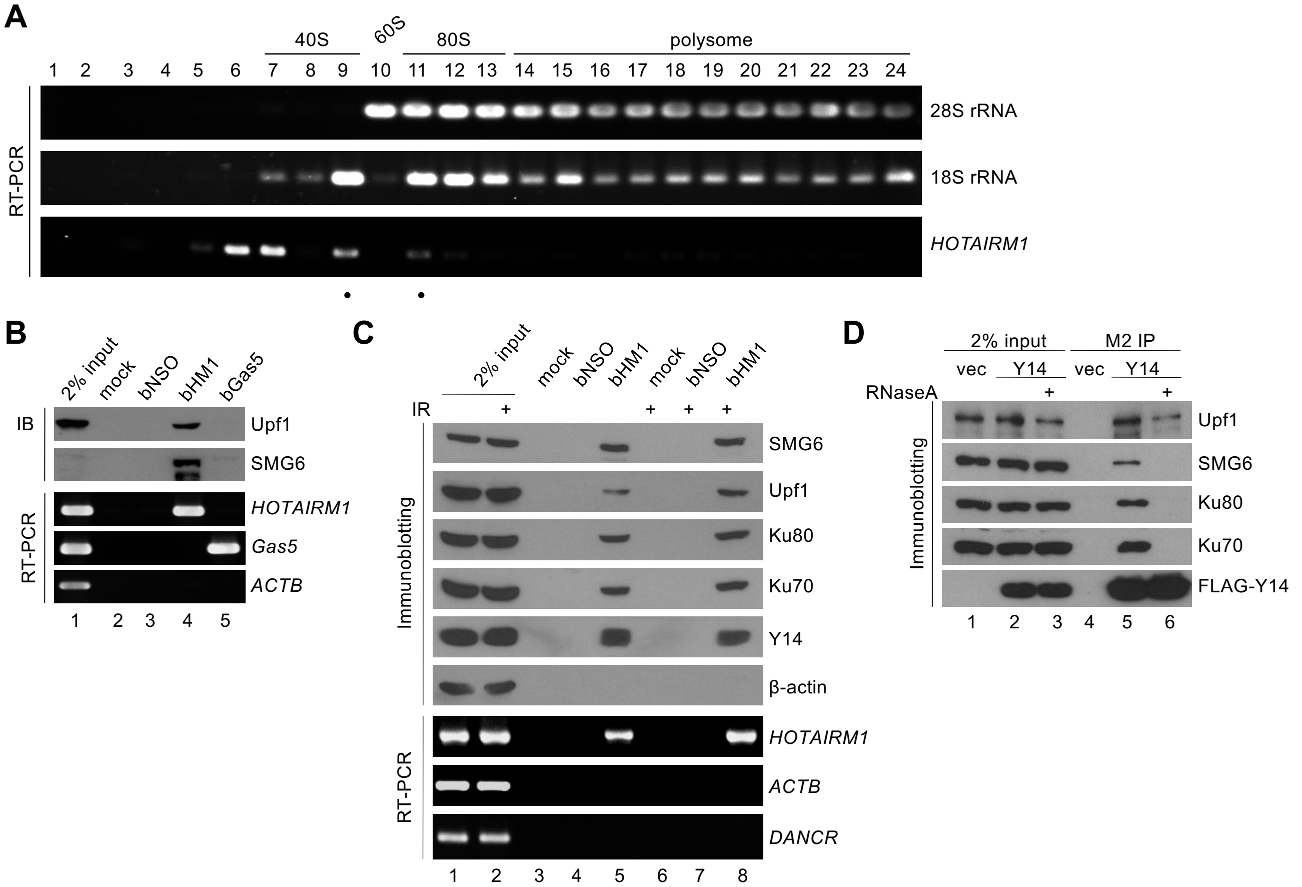
A
40S
60S
80S
polysome
1 2 3 4 5 6 7 8 9 10 11 12 13 14 15 16 17 18 19 20 21 22 23 24
28S rRNA
18S rRNA
HOTAIRM1
RT-PCR
B
2% input
mock
bNSO
bHM1
bGas5
IB
Upf1
SMG6
RT-PCR
HOTAIRM1
Gas5
ACTB
1 2 3 4 5
C
2% input
mock
bNSO
bHM1
mock
bNSO
bHM1
IR
+
+
+
+
Immunoblotting
SMG6
Upf1
Ku80
Ku70
Y14
β-actin
RT-PCR
HOTAIRM1
ACTB
DANCR
1 2 3 4 5 6 7 8
D
2% input
M2 IP
vec
Y14
vec
Y14
RNaseA
+
+
Immunoblotting
Upf1
SMG6
Ku80
Ku70
FLAG-Y14
1 2 3 4 5 6

FigS7

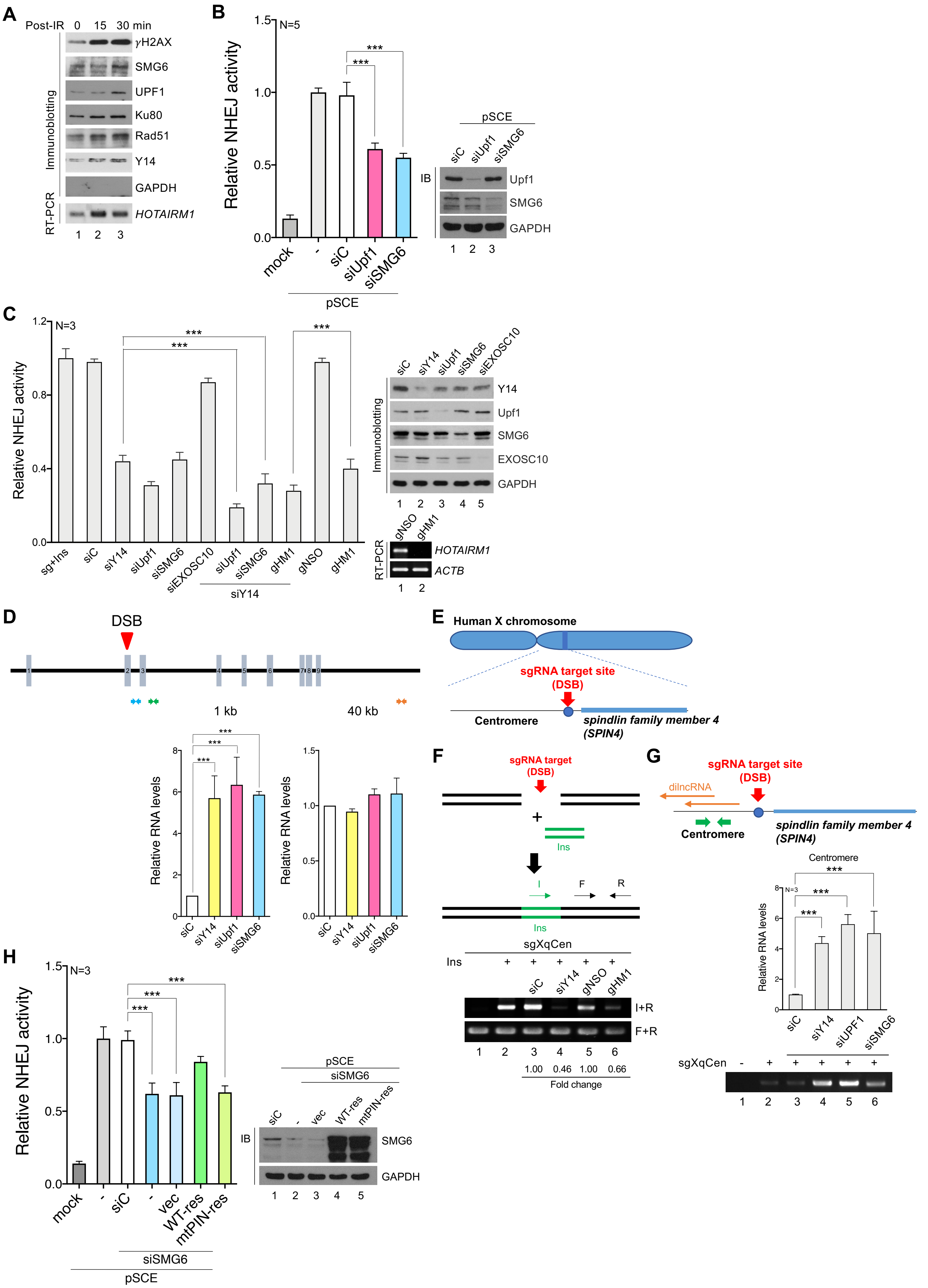

A
Post-IR 0 15 30 min
γH2AX
SMG6
UPF1
Ku80
Rad51
Y14
GAPDH
HOTAIRM1
Immunoblotting
RT-PCR
1 2 3
B
N=5
Relative NHEJ activity
mock
-
siC
siUpf1
siSMG6
pSCE
pSCE
siC siUpf1 siSMG6
IB
Upf1
SMG6
GAPDH
1 2 3
C
N=3
Relative NHEJ activity
sg+Ins
siC
siY14
siUpf1
siSMG6
siEXOSC10
siY14
gHM1
gNSO
siC siY14 siUpf1 siSMG6 siEXOSC10
Y14
Upf1
SMG6
EXOSC10
GAPDH
Immunoblotting
1 2 3 4 5
gNSO gHM1
RT-PCR
HOTAIRM1
ACTB
1 2
D
DSB
1 kb
40 kb
Relative RNA levels
siC
siY14
siUpf1
siSMG6
E
Human X chromosome
sgRNA target site
(DSB)
Centromere
spindlin family member 4
(SPIN4)
F
sgRNA target
(DSB)
Ins
I
F
R
sgXqCen
Ins
siC siY14 gNSO gHM1
I+R
F+R
1 2 3 4 5 6
1.00 0.46 1.00 0.66
Fold change
G
sgRNA target site
(DSB)
dilncRNA
Centromere
spindlin family member 4
(SPIN4)
Centromere
N=3
Relative RNA levels
siC
siY14
siUPF1
siSMG6
sgXqCen
1 2 3 4 5 6
H
N=3
Relative NHEJ activity
mock
-
siC
vec
WT-res
mtPIN-res
siSMG6
pSCE
pSCE
siSMG6
siC - vec WT-res mtPIN-res
IB
SMG6
GAPDH
1 2 3 4 5

Figure S8

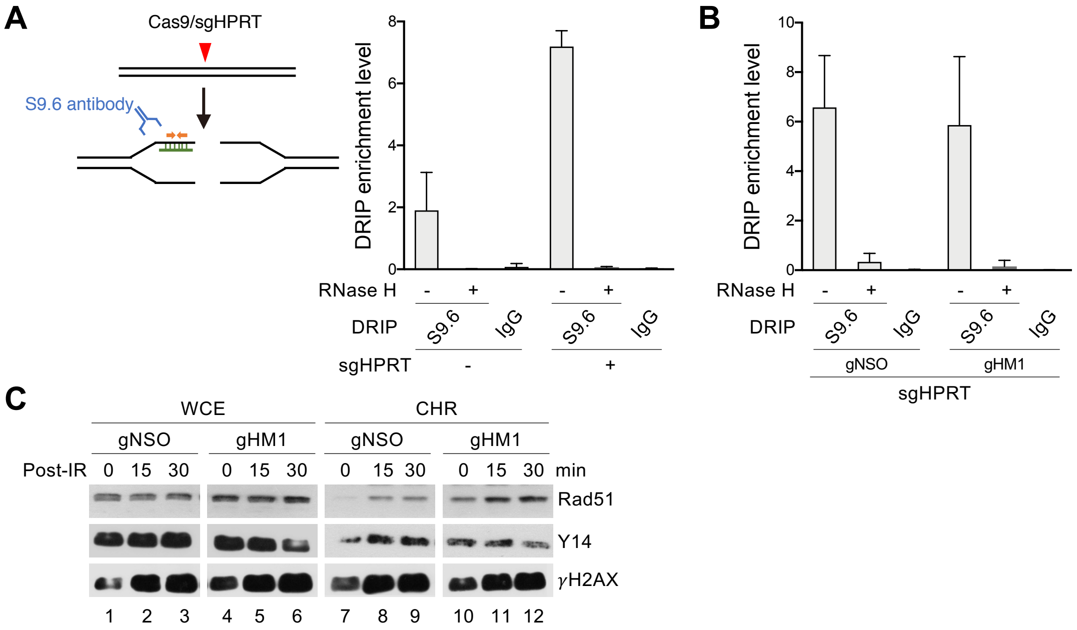
A
Cas9/sgHPRT
S9.6 antibody
DRIP enrichment level
RNase H
DRIP
S9.6
IgG
sgHPRT
B
DRIP enrichment level
RNase H
DRIP
S9.6
IgG
gNSO
gHM1
sgHPRT
C
WCE
CHR
gNSO
gHM1
Post-IR 0 15 30 0 15 30 0 15 30 0 15 30 min
Rad51
Y14
γH2AX
1 2 3 4 5 6 7 8 9 10 11 12

Table S7: List of Antibodies

Mouse monoclonal anti-GAPDH	ProteinTech
Mouse monoclonal anti-β-actin	ProteinTech
Mouse monoclonal anti-γH2AX	Millipore
Mouse monoclonal anti-S9.6	Millipore
Mouse monoclonal anti-NBS1	GeneTex
Mouse monoclonal anti-Lamin A/C	Sigma
Rabbit polyclonal anti-FLAG epitope	Sigma
Rabbit polyclonal anti-Y14	GeneTex
Rabbit polyclonal anti-SMG6	GeneTex
Rabbit polyclonal anti-XLF	GeneTex
Rabbit polyclonal anti-XRCC4	GeneTex
Rabbit polyclonal anti-His	GeneTex
Rabbit polyclonal anti-SMG1	Cell Signaling Technology
Rabbit polyclonal anti-Ku70	Cell Signaling Technology
Rabbit polyclonal anti-Ku80	Cell Signaling Technology
Rabbit polyclonal anti-Upf1	Cell Signaling Technology
Rabbit polyclonal anti-H3	Cell Signaling Technology
Rabbit polyclonal anti-DNA ligase IV	ProteinTech
Rabbit polyclonal anti-HA	Bioman
Rabbit polyclonal anti-γH2AX	Novus
Rabbit polyclonal anti-53BP1	Novus
Rabbit polyclonal anti-Biotin	Bethyl
Rabbit polyclonal anti-Rrp6/EXOSC10	Sigma
Rabbit polyclonal anti-Rad51	Abcam
HRP-conjugated anti-rabbit IgG	GE Healthcare
HRP-conjugated anti-mouse IgG	Thermo Fisher Scientific
FITC-conjugated anti-mouse IgG	Cappel
Rhodamine-conjugated anti-mouse IgG	Cappel
Alexa Fluor 568-conjugated anti-rabbit IgG	Thermo Fisher Scientific
Texas Red-conjugated anti-Biotin	Rockland

Table S8. List of Oligonucleotides

GGGUAUACUCUAGUUGAAUAUGAAA	Thermo Fisher Scientific	siY14
UAACGUUAGUAAGGUUAGG	Thermo Fisher Scientific	siLIG4
CCCAACCCGAUAAACCGAUGUUCU	Thermo Fisher Scientific	siUpf1
UACUUCAGCACUGUGACCC	Thermo Fisher Scientific	siSMG6
GGAUCGAAGUAAAGUGACU	Thermo Fisher Scientific	siRrp6/EXOSC10
CCCAAUUCAGCAGCAUAUUUGGAAU	Thermo Fisher Scientific	siKu80
AACACGTCTATACGC	QIAGEN	gNSO
ACTAGGGCTTCTAATA	QIAGEN	gHM1
Biotin-TAAAATACCATTTGATGTTTGAAATTAT	GENOMICS	bNSO
Biotin-ATCAACAATTCATTTGTTTATACAC	GENOMICS	bHM1-1
Biotin-GCGATGTTTCTTCATAGGTTTGCTC	GENOMICS	bHM1-2
Biotin-ATCAACAATTCATTTGTTTATACAC	GENOMICS	bHM1-3
Biotin- GGCACACAGGCATTAGACAGAAA	GENOMICS	bGas5
TAAAATACCATTTGATGTTTGAAATTAT	Mission Biotech	NSO
CAGTTCATCTTTCATTGAACGGTG	Mission Biotech	AS1
CATTTATTAAGGCGGCATGTTCAAAG	Mission Biotech	AS2
TTAATTGAGTTGTCATATGTTAATAACCGG	Mission Biotech	dsDNA-1 (Ins)
ACCGTTATTAACATATGACAACTCAATTA	Mission Biotech	dsDNA-2 (Ins)
CTAATCATTATGCTGAGGATTTGGGTTTAATTGA GTTGTCATATGTTAATAACGGTATACTAATTATG GACAGGTAAGTAAGATCTTAAAATGAGGTTTTT TAC	GENOMICS	ssODN
CACCGTTCAATGAAAGATGAACTG	Mission Biotech	HOTAIRM1 (forward)
CATTTATTAAGGCGGCATGTTCAAAG	Mission Biotech	HOTAIRM1 (reverse)
GAAAATCTGGCACCACACCT	Mission Biotech	ACTB (forward)
GGCCGGACTCGTCATACTC	Mission Biotech	ACTB (reverse)
GCAGGTTCCGGGAGGTCA	Mission Biotech	SNHG25 (forward)
CAAACCACTTTATTGACGGGAA	Mission Biotech	SNHG25 (reverse)
GACTGCAGACCCCTAACCTT	Mission Biotech	SNHG9 (forward)
ACCCGCATGCAGTGAGTTA	Mission Biotech	SNHG9 (reverse)
TAGAGATGCAAAGATACACGAAA	Mission Biotech	SNHG5 (forward)
CCAAGACAATCTGGCCTCTATC	Mission Biotech	SNHG5 (reverse)
TGGATGCTGTCAGCTAAGTTCAC	Mission Biotech	CRNDE (forward)
TTCCAGTGGCATCCTCCTTATC	Mission Biotech	CRNDE (reverse)
GCGTCCGAAGTATGAGTCCA	Mission Biotech	JPX (forward)
GGCGATCAGCGAGAAAGAA	Mission Biotech	JPX (reverse)
AGGTAGAACCTCTATGCATTTTGTG	Mission Biotech	TUG1 (forward)
ACTCTTGCTTCACTACTTCATCCAG	Mission Biotech	TUG1 (reverse)
GCTGGAGTTGCGCGGGCTGACG	Mission Biotech	DANCR (forward)
GGGTGTAATCCACGTTTCTCAT	Mission Biotech	DANCR (reverse)
GATGCCTGATCTCATCAATCTAG	Mission Biotech	DLEU2 (forward)
AGGCTGTTCTCCAGAATTGG	Mission Biotech	DLEU2 (reverse)
TAATACGACTCACTATAGGG	Mission Biotech	MS2 (forward)
TAGAAGGCACAGTCGAGG	Mission Biotech	MS2 (reverse)
ATCCAATCAAATGTTTGTATCCTGT	Mission Biotech	HPRT (forward)
GAGTTGTCATATGTTAATAACGG	Mission Biotech	HPRT (Ins)
CCCTTCAATGTTTACTTTGTTCTGG	Mission Biotech	HPRT (reverse)
CAGAGCAAGACTCTGTCTC	Mission Biotech	DSB upstream (forward)
CTCCTGACCTCAGGTGATCCAC	Mission Biotech	DSB upstream (reverse)
CACTGGCTATGCATGTATAC	Mission Biotech	DSB downstream (forward)

GATAGCACTAGACACAC	Mission Biotech	DSB downstream (reverse)
GCAGTGGTTCTTGACGGGTA	Mission Biotech	ALMS1-IT1 (forward)
CATTACAAGTATTGAGACAAATC	Mission Biotech	ALMS1-IT1 (reverse)
GAGGTAGGAGTCGACTCCTGTGAG	Mission Biotech	Gas5 (forward)
GTTACCAGGAGCAGAACCATTAAG	Mission Biotech	Gas5 (reverse)
TACCACATCCAAGGAAGGGAGCA	Mission Biotech	18S rRNA (forward)
TGGAATTACCGCGGCTGCTGGCA	Mission Biotech	18S rRNA (reverse)
AACGAGATTCCCACTGTCCC	Mission Biotech	28S rRNA (forward)
CTTCACCGTGCCAGACTAGAG	Mission Biotech	28S rRNA (reverse)
ATTGAAGCCAAGAAAAAGGATC	Mission Biotech	Ku80 deletion (forward)
AATCGATTTATAGGCTGCAATCC	Mission Biotech	Ku80 deletion (reverse)
AAAGGGTGTTTATTCCTCA	Mission Biotech	sgHPRT (forward)
CTCGTGCTTCTTATCCTCTTC	Mission Biotech	sgHPRT (reverse)
CAGAGCAAGACTCTGTCTC	Mission Biotech	DRIP DSB upstream (forward)
CTCCTGACCTCAGGTGATCCAC	Mission Biotech	DRIP DSB upstream (reverse)
GTCAAACATAACTGTGTGCGC	Mission Biotech	sgXqCen DSB upstream (forward)
GACCTTCTGTCTTTCGTCTACC	Mission Biotech	sgXqCen DSB upstream (reverse)
GCATCGACAACAGCTAAGTG	Mission Biotech	sgXqCen DSB downstream (forward)
AACCGTGAGCACTGGGACAG	Mission Biotech	sgXqCen DSB downstream (reverse)
GCCGTCTAGGTGACG CGCAGTCGC	Mission Biotech	HOTAIRM1 exon1 (forward)
CAGTTCATCTTTCATTGAACGGTG	Mission Biotech	HOTAIRM1 exon1 (reverse)
ACATCGCGTTGTCATTGGAA	Mission Biotech	HOTAIRM1 exon2 (forward)
TGGGTTCAGGCAAAACAGAC	Mission Biotech	HOTAIRM1 exon2 (reverse)
GAACCCATCAACAGCTGGGAGATTAATCAA	Mission Biotech	HOTAIRM1 exon3 (forward)
GAGGCAGAATTGGACAGTCTAAGATTTGGGCCA	Mission Biotech	HOTAIRM1 exon3 (reverse)
GTCTGTTTTGCCTGAACCCATC	Mission Biotech	HOTAIRM1 OE (forward)
CTACAGCTAGAGCTAGAC	Mission Biotech	HOTAIRM1 OE (reverse)
GAAGTAAAGTGATAAGATATGG	Mission Biotech	DSB downstream 1K (forward)
GAATACCCGTATGTTCATCAC	Mission Biotech	DSB downstream 1K (reverse)
GTGGACTCTAATCCGATATG	Mission Biotech	DSB downstream 40K (forward)
GTTCTGGCGACTAGAAGTCCAAG	Mission Biotech	DSB downstream 40K (reverse)
